# Describing the Core Attributes and Impact of Comprehensive Cancer Centers Internationally: A Chronological Scoping Review

**DOI:** 10.3390/cancers17061023

**Published:** 2025-03-18

**Authors:** Carla Thamm, Elise Button, Jolyn Johal, Reegan Knowles, Catherine Paterson, Michael T. Halpern, Andreas Charalambous, Alexandre Chan, Sanchia Aranda, Carolyn Taylor, Raymond J. Chan

**Affiliations:** 1 Caring Futures Institute, College of Nursing and Health Sciences, Flinders University, Adelaide, SA 5042, Australia; carla.thamm@flinders.edu.au (C.T.);; 2 Cancer and Palliative Care Outcomes Center, School of Nursing, Queensland University of Technology, Brisbane, QLD 4059, Australia; 3 Central Adelaide Local Health Network, Adelaide, SA 5000, Australia; 4 Department of Health Policy and Health Services Administration, School of Public Health, University of Texas Health Science Center, San Antonio, TX 78229, USA; 5 Faculty of Health Sciences, Cyprus University of Technology, 3036 Limassol, Cyprus; andreas.charalambous@cut.ac.cy; 6 Faculty of Health Sciences, University of Turku, 20014 Turku, Finland; 7 School of Pharmacy and Pharmaceutical Sciences, College of Health Sciences, University of California, Irvine, CA 92697, USA; 8 Medicine, Dentistry and Health Sciences, University of Melbourne, Melbourne, VIC 3010, Australia; 9 Global Focus on Cancer, South Salem, NY 10590, USA; carolyn@globalfocusoncancer.org

**Keywords:** attributes, chronological, comprehensive cancer center, impact, international, scoping review

## Abstract

**Background/Objectives**: Comprehensive cancer centers (CCCs) remain at the forefront of cancer control efforts. Limited clarity and variation exist around the models, scope, characteristics, and impacts of CCCs around the globe. This scoping review systematically searched and synthesized the international literature, describing core attributes and anticipated and realized impacts of CCCs, detailing changes over time. **Methods**: Searches for English language sources were conducted across PubMed, Cochrane CENTRAL, Epistemonikos, and the gray literature from January 2002 to April 2024. Data were extracted and appraised by two authors. Results were narratively synthesized. **Results**: Of 3895 database records and 843 gray literature sources screened, 81 sources were included. Papers were predominantly opinion-based, from the USA and Europe, and published between 2011 and 2020. Internationally, the interconnected attributes of CCCs included (1) clinical service provision; (2) research, data, and innovation; (3) education and clinical support; (4) networks and leadership; (5) health equity and inclusiveness; and (6) accountability and governance. Largely anticipated impacts were synergistic and included delivery of optimal, person-centered, complex care; development of a highly qualified cancer workforce; greater research activity and funding; effective, strategic alliances; and reduction in cancer-related inequalities. Limited evidence was found demonstrating measurable broad outcomes of CCCs. The early literature highlighted the establishment, development, and accreditation of CCCs. The ongoing literature has reflected the evolution of cancer care, key areas for growth, and limitations of CCCs. Recently, the CCC literature has increased exponentially and focused on the need for CCCs to drive networks and leadership to address health equity and inclusiveness. **Conclusions**: Results suggest that CCCs are yet to reach their full potential, with future efforts ideally focusing on accountability, effective networking, and health equity at a local, national, and international level. CCCs must generate evidence of impact, and continue to evolve in line with contemporary healthcare, to fulfil their role in cancer control efforts.

## Simple Summary

Comprehensive cancer centers are an important component of cancer control efforts, that have evolved over time. Significant variation exists internationally in the setting, context, and healthcare models in which they operate. Greater clarity is needed regarding the defining characteristics and core functions of comprehensive cancer centers that distinguish them from other types of cancer centers, to inform accreditation programs. The potential impact of comprehensive cancer centers at the patient, provider, organization, system, and societal levels must also be understood to justify the development and continued support, and inform measurements of success. The findings of this chronological scoping review are valuable as they inform refinement and development of comprehensive cancer centers and cancer control efforts, highlighting key priority areas for that require future focus.

## 1. Introduction

Comprehensive cancer centers (CCCs) were first recognized in 1973 by the National Cancer Institute (NCI) in the USA, with the intended purpose of bringing research findings to the greatest number of people as quickly as possible [1]. Development and support for CCCs was established under the National Cancer Act of 1971, which represented the USA’s commitment to the “war on cancer”—focused largely on supporting cancer research and training and supporting cancer researchers in NCI-designated CCCs [1]. In 2002, the World Health Organization (WHO) recommended that adequately resourced countries enforce the development and networking of comprehensive cancer treatment centers as a priority action in national cancer control programs [2]. The WHO outlined that these centers should be active in clinical training and research, and serve as national and international reference centers [2]. In 2008, the Organization for European Cancer Institutes (OECI) launched a program to recognize CCCs, based on an adaptation of the NCI accreditation methodology, while the German Cancer Society in partnership with the German Cancer Aid also started their own certification program for CCCs in Germany in 2007 [3,4,5]. Over time, a number of CCCs have been established globally, predominantly in high-income countries [6]. While the original intention of creating CCCs is still relevant, new CCCs may have aims and functions that have evolved over time.

Comprehensive Cancer Centers are defined broadly as centers of excellence in cancer care, research, and education, based on a multiprofessional, interdisciplinary, and multispecialty paradigm [6]. They are recognized as the highest tier of cancer centers and are reported to provide comprehensive care across the cancer continuum (including prevention), drive research and innovation, and be leaders in national cancer control efforts [7]. CCCs can consist of a center or a network of national or regional infrastructures providing services [8]. Variation exists internationally in the availability, purpose, role, characteristics, challenges, and opportunities of CCCs, which have evolved in line with changing burden of disease, demographics, growing social expectations, increasing inequalities, and scarcity of resources [9]. In Europe, the current recommendations are for one CCC per 5–10 million people [10]. The USA and Germany have approximately one CCC per 5–6 million people [7,11]. Other countries are in the early stages of developing CCCs, and some countries have yet to establish a single CCC [3]. However, the mere presence of a CCC does not guarantee equitable high-quality cancer care for all [12,13].

Although CCCs are reported to be an important component of cancer control efforts, significant variation exists internationally in the cancer center models in which they operate [9]. Greater clarity is needed regarding the defining characteristics and core functions of CCCs that distinguish them from other types of cancer centers. The potential impact of CCCs at the patient, provider, organization, system, and societal levels must also be understood to justify the development and continued support of CCCs and inform measurements of success. Many lessons can be learned from countries with well-established models of CCCs, around successes, limitations, and future directions [9]. To the best of our knowledge, no review has collated, described, and synthesized the broad international published and unpublished literature on the attributes and impacts of CCCs. The aim of this high-level review was to describe the defining characteristics of CCCs, their anticipated and realized impact, and changes to the CCC literature over time. Specifically, the review questions were as follows: (1) what are the key attributes of CCCs; (2) what are the anticipated and realized impacts of CCCs; and (3) how has the CCC literature evolved over time?

## 2. Materials and Methods

A chronological scoping review was conducted with a systematic search, exploring the attributes and impacts of CCCs as they have evolved over time. The JBI (Joanna Briggs Institute) methodology [14] and PRISMA guidelines (Preferred Reporting Items for Systematic Reviews and Meta-Analyses extension for Scoping Reviews PRISMA-ScR checklist [15]) (Supplementary File S1) were followed. The protocol for this scoping review and a complimentary concurrent systematic review was registered in one protocol prospectively with the Prospective Register of Systematic Reviews database (PROSPERO ID# CRD42023387620).

### 2.1. Search Strategy

Searches were conducted across the following databases: PubMed, Cochrane Library, CINAHL (EBSCOhost), and Epistemonikos. Gray literature searches (for information sources that are not commercially published) were also conducted through PROSPERO International Prospective Register of Systematic Reviews, Google, Google Scholar, and World Health Organization Institutional and Repository for Information Sharing. Reference lists of included sources were searched to identify further potentially relevant articles. Library catalogues were not searched; however, book chapters were found in database, gray literature, and hand searching. Cross-checking was performed between the current scoping review and a concurrent systematic review (conducted by the authorship team) exploring differences in outcomes between CCCs and non-CCCs [16]. Search terms used were centered on “comprehensive cancer center”. Searches were conducted in January 2023, and repeated in October 2023 and May 2024. The full search strategy can be found in Supplementary File S2.

### 2.2. Eligibility

Eligibility criteria (Table 1) included any source in English that provided information on the key attributes or impacts (anticipated or realized) of CCCs in any country. Sources published from January 2002 to May 2024 were included to coincide with the year the WHO National Cancer Control Programs Policies and Managerial Guidelines were published, calling for the reinforcement of the development of CCCs internationally [2]. In countries with accreditation and designation programs, sources focused on centers formally designated as CCCs were included. In countries without formal accreditation programs, sources reporting on self-declared CCCs were included.

### 2.3. Article Selection

Records retrieved from the database searches were imported into Covidence software, with duplicates removed. Two reviewers independently screened the title, abstract, and full text (CT and EB) against eligibility criteria. The gray literature was searched by two researchers independently (EB and JJ), and sources were assessed against the selection criteria by one author and reviewed by a second author. Discrepancies were resolved through discussion until consensus was reached. All decisions were recorded in study-specific tables.

### 2.4. Data Extraction and Synthesis

Characteristics of the individual sources (study design, description of source, country, setting, author, and cancer type), along with descriptions of the attributes and impacts of CCCs, were extracted into a Microsoft Word data extraction form (developed specifically for the review) chronologically by one author (EB) and checked for accuracy by a second author (CT). Discrepancies were resolved through discussion until consensus was reached. Where there were missing data, the authors contacted the corresponding authors of the relevant articles for more information.

Findings were synthesized via narrative analysis [17] overall, and according to decade of publication (2002–2009, 2010–2019, 2020–2024) by doctorally prepared researchers each with over 20 years of clinical experience (CT, EB). Data on attributes and impacts of CCCs were extracted from the text of included sources and coded, categorized, and themed inductively [18]. Themes relating to attributes and impacts of CCC were synthesized to understand interrelated concepts. Within the analysis, focus was placed on identifying the defining features of CCCs and the anticipated versus realized impacts, and how they have evolved over time (from the earliest available evidence onwards). Quality assessment was not conducted as is standard in scoping reviews.

## 3. Results

### 3.1. Included Sources

**Figure 1:**
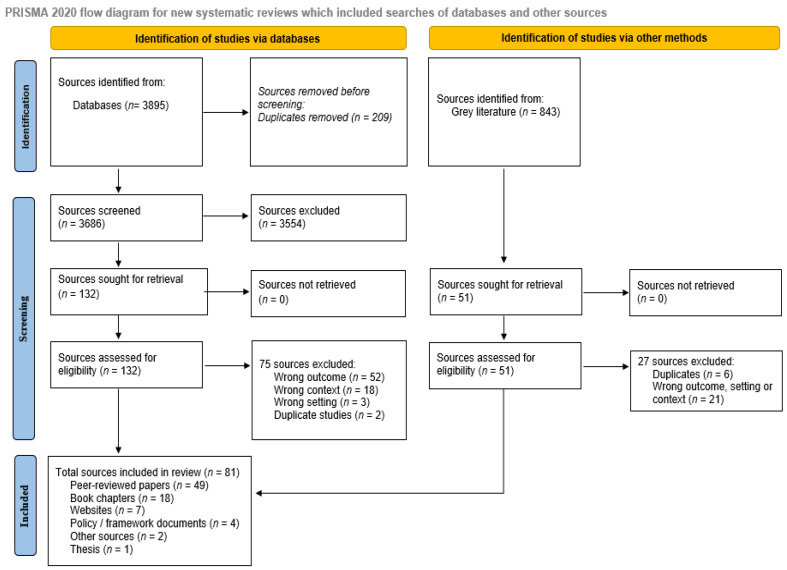
Scoping review PRISMA flow diagram.

Of the 3895 records identified from databases and 843 records from the gray literature, a total of 81 sources were included (Figure 1).

Sources included 49 peer-reviewed articles [3,4,5,10,19,20,21,22,23,24,25,26,27,28,29,30,31,32,33,34,35,36,37,38,39,40,41,42,43,44,45,46,47,48,49,50,51,52,53,54,55,56], 18 book chapters (in two books) [57,58,59,60,61,62,63,64,65,66,67,68,69,70,71,72,73,74], 7 websites [11,75,76,77,78], 4 policy/framework documents [79,80,81], 2 white papers [82,83], and 1 thesis [84] (Table 2). The 49 peer-reviewed articles consisted of 26 opinion pieces, commentaries, or reviews [5,10,26,30,34,35,36,38,39,42,45,47,48,50,54], 13 observational studies [20,52,53], and 10 mixed methods or qualitative studies [3,4,5,10,11,21,22,23,25,27,28,29,30,31,32,33,36,37,40,41,45,46,49,51,76,77,79,81,82,84]. Most studies (*n* = 42, 56%) were published from 2020 onwards [3,10,13,40,42,43,44,45,47,49,50,52,53,54,56,57,58,59,60,61,62,63,64,65,66,67,68,69,70,71,72,73,81,82,85,86,87,88,89,90,91].

The purpose of the sources varied widely: 21 (17%) provided practical guidance on development of CCCs; [57,58,59,60,61,62,63,64,65,66,67,68,69,70,71,72,73,74,80] 17 (14%) described characteristics, services, or practices of CCCs [10,24,26,27,30,39,43,44,50,51,53,74,80,89,90,91,94]; 10 (8%) reported on clinical service provision in CCCs [10,24,25,26,27,30,40,41,43,44,48,50,51,53]; and nine (7%) focused on vision and roles of CCCs [20,34,35,38,39,42,45,47,49]. Supplementary File S3 displays characteristics of included sources. Sources were predominantly focused on CCCs in Europe (*n* = 34, 42%) [3,4,5,10,11,13,21,22,23,25,27,28,29,30,31,32,33,36,37,40,41,45,46,49,51,76,77,79,81,82,84,85,87,88], or USA (*n* = 23, 28%) [20,24,34,35,38,39,42,43,44,47,48,50,52,53,54,55,56,75,78,89,90,91,93]. Four sources focused on low- or middle-income countries including: a network between CCCs in a high- and middle-income country (UK and India) [19]; a CCC model within a low-income country (Africa) [94]; guidance on development of CCCs in countries with limited resources [65]; and description of a global program where CCCs in the USA support cancer control in low- and middle-income countries [93].

### 3.2. Attributes and Impacts of CCCs

Key attributes and anticipated and/or realized impacts of CCCs were identified under the following themes: (1) clinical service provision; (2) research, data, and innovation; (3) education and clinical support; (4) networks and leadership; (5) health equity and inclusiveness; and (6) accountability and governance. Table 3 displays the attributes and impacts of CCCs under the key themes, as reported across all sources (*n* = 81) (see Supplementary File S4 for more detail).

Table 4 provides a summary of the characteristics of current CCC accreditation and designation programs.

### 3.3. Synthesis of Attributes and Impacts of CCCs

**Figure 2:**
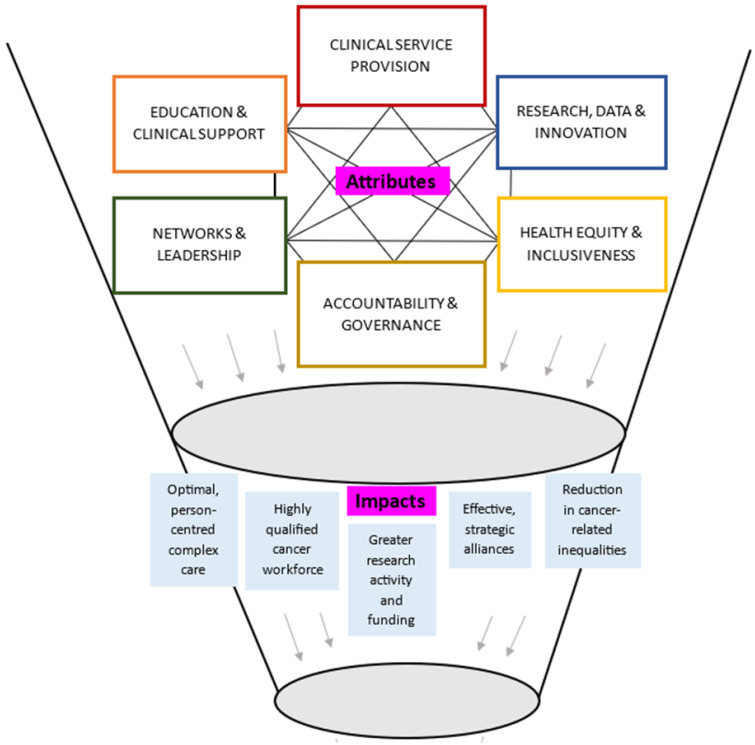
Theoretical model synthesizing attributes and impacts of CCCs.

Figure 2 displays a theoretical model of the key attributes and anticipated impacts of CCCs, informed by narrative synthesis of the largely opinion-based included sources. The synthesis highlighted that the core attributes of CCCs were likely interconnected and interdependent. An example of this was that high-quality clinical service provision could be underpinned by excellence in research and innovation. CCCs were reported to conduct clinically relevant research from the bench-to-bedside, and back again through multispecialty and multidisciplinary teams, and access to high patient numbers across the cancer trajectory. This research was then able to be directly translated into practice via education and clinical support, both within the CCC, and within local, national, or international networks. Networks and leadership could enable collaboration and sharing of resources to support research and innovation, education and clinical support, and health equity and inclusiveness, by addressing needs of the local population and focusing on rare types of cancers. Excellent standards across the attributes could be supported via accountability and governance, particularly through accreditation and designation programs.

Synergistic impacts of CCCs were reported to be mostly aligned with core attributes. Optimal person-centered complex care could be enabled by a highly qualified cancer workforce. Effective and strategic alliances could lead to greater research activity and funding and improve cancer-related outcomes. Figure 2 does not represent all possible reported or anticipated impacts (see Table 3 for a breakdown of attributes and impacts of CCCs).

### 3.4. Primary Research

Table 5 outlines findings in the 24 sources that reported observational primary research, which focused on health equity (grant schemes, community engagement, and access to care) (*n* = 7) [34,35,38,39,42], availability of clinical services in CCCs (*n* = 6) [26,30,34,35,45,47], website content of CCCs (*n* = 3) [38,39,42], secondary analyses of accreditation data (*n* = 3) [5,10,53], and benefits of second opinions at CCCs (*n* = 2) [52,85]. Of note, one study reported no difference in cancer prevention measures in designated CCCs compared to non-CCCs [10], despite several sources stating that cancer prevention was a core concern of CCCs [13,28,80,88]. All observational studies were descriptive, with the exception of five exploratory studies, which reported CCCs were associated with greater academic output [48], higher rates of participation in clinical trials [91], and improved treatment plans in second opinions [52], but lack of equitable access to care [55,56].

### 3.5. Changes in the CCC Literature over Time

Table 6 provides a summary of the CCC literature in various time periods for each of the themes. The few sources (*n* = 4) published between 2002 and 2009 described early establishment of CCCs [26], initiation of accreditation and designation programs [4], and strategies for CCCs to align with cancer control programs [79]. The literature from 2010 to 2019 (*n* = 28) explored clinical service provision of CCCs [20,25,34,35] (with a focus on supportive and integrative care services) [27,34,38,39] and research opportunities for CCCs (between CCC networks, and greater focus on translational research) [48]. The literature on the growth and development of networks (between CCCs, and CCCs and community organizations) [19,22,33,37] and accreditation and designation programs [5,21,29,31,32], and barriers in equitable access to care, were also reported in 2010–2019. Recently (2020–2024), the literature on CCCs has increased exponentially (*n* = 42) and focused on a range of issues across all identified attributes of CCCs. The recent literature has highlighted the need for CCCs to focus on networks and leadership, to address health equity and inclusiveness (Table 6).

## 4. Discussion

This review reports findings of a comprehensive search and synthesis of 81 published and unpublished sources describing the key attributes and (largely anticipated) impact of CCCs, and chronological changes in the CCC literature. The evolution of the CCC literature has reflected the progress of CCCs over time; from articulation of vision; to development of centers (and larger scale deployment of CCC), services, systems, and programs; and to a focus on areas for improvement. Changes in the CCC literature have also reflected an increasing focus on supportive and integrative care, in line with a greater understanding of the benefits of such services and the recognition of cancer as a chronic illness [1,2,81]. The most notable development in the CCC literature was a recognition of issues surrounding health equity, and subsequent development of strategies to address the issue at a local, national, and international level. This work is significant, timely, and can inform the development and improvement of CCCs within particular health systems internationally, which are regarded as vital in addressing the burden of cancer globally [1,2,81]. Most sources were opinion pieces and therefore findings must be interpreted through this lens.

### 4.1. Attributes and Impacts

Key, interdependent attributes of CCCs were found across six themes: (1) clinical service provision; (2) education and clinical support; (3) research, data, and innovation; (4) health equity and inclusiveness; (5) networks and leadership; and (6) accountability and governance (Figure 2). While many of these attributes are accepted as core components of comprehensive cancer care and substantiate and build upon the WHO-IAEA Framework [96], the symbiotic relationship of these attributes in CCCs is yet to be fully explored. The literature indicates that CCCs serve as a nexus, where the core attributes of CCCs are intimately linked and needed for CCCs to reach their full potential. Evidence is lacking on the importance of having all attributes present within a standalone CCC, or if such attributes can successfully be provided within a networked approach. Although reported as ideal [4,26], the presence of all attributes in a single physical location may not be feasible or realistic in many countries, requiring networking and alliances of infrastructure and services [83]. The concept of CCCs has evolved from standalone CCCs, to include approaches with CCCs as core elements within comprehensive cancer networks—with an increased obligation on the CCC to drive improvements of care for all [92]. Our findings suggest that the success of CCCs lies in having all six attributes present in some form (potentially drawing on a networked approach), to produce synergistic impacts both within and beyond the CCC.

The literature describes the ambitious goals set out for CCCs, often aligned with the objectives of national or international cancer control plans [1,2,81]. These goals included providing equal access to high-quality cancer care [4,76], education, support, and training for cancer clinicians beyond the CCC, to foster and accelerate transdisciplinary state-of-the-art clinical research, and translation across the cancer trajectory [1,4,76]. In the US, the initial purpose of CCCs was to bring research findings to the greatest number of people as quickly as possible [1]. Our findings highlight the synergistic impacts of CCCs that were anticipated to flow on from core attributes of CCCs. Largely opinion-based sources reported that CCCs can lead to a broad range of positive impacts, including delivery of optimal, person-centered, complex care; a highly qualified cancer workforce; greater research activity and funding; effective, strategic alliances; and reduction in cancer-related inequalities. A framework is needed to assess the impacts of CCCs and justify current and future investment.

Most sources in this review were set in countries that participated in accreditation and designation programs that subjectively assessed the presence of attributes and quality markers of CCCs. Accreditation criteria were viewed as valuable as they defined the essential components and prescribed standards, distinguishing CCCs from other types of cancer centers [1,11,76,77,82]. The impacts of accreditation and designation programs were reported to include defining excellence [11,75,76], increased academic output of clinical staff [48], identification of strengths and weaknesses of a center to inform improvement efforts [36], and greater collaboration between designated centers [11,75,76]. While the USA and European countries have long-standing, robust accreditation, and designation systems for CCCs [7,97], this is not the case in all countries with CCCs [26,92]. It is acknowledged that formal (or mandatory) accreditation and designation programs may not be practical, feasible, or desired in all countries. We emphasize it is vital for all CCCs to have key performance indicators within systems of accountability and governance, to define the attributes of CCCs, benchmark outcomes, promote standards of excellence, and define the role of CCCs within the wider provision of cancer services.

The anticipated impacts of CCCs are well described in the international literature, but to date, are largely unsubstantiated in empirical research. The 24 peer review studies in this review largely reported descriptions of availability of clinical services and patient resources [20,34,35,38,42,45,47,85]. Although three primary research studies reported observed benefits associated with CCCs [48,52,91], two studies reported CCCs were associated with inequitable access to care [55,56]. In relation to the original intent of CCCs in the USA, unanswered questions remain in the peer-reviewed literature regarding 1) the extent that investment in discovery and testing of new treatments in CCCs leads to widescale spread through engagement between CCCs and external organizations; and 2) the wider impact of CCCs on cancer outcomes for the population. More research is desperately needed exploring the impact of CCCs, particularly within different government healthcare funding models, to guide their role within the overall health system. A recent systematic review of patient-relevant outcomes, conducted by the authorship team, reported superior mortality and survival, and quality of care outcomes, in CCCs compared to non-CCCs [16]. Studies reporting health equity and cost outcomes favored non-CCCs over CCCs, and there was a dearth of literature focused on symptoms, health-related quality of life, treatment experience, and economic evaluation [16]. Future research is needed to understand if the goals of CCCs are being realized, and if this leads to positive impacts at a societal, organizational, provider, and patient level.

### 4.2. Opportunities and Drivers for Change

The results of this review suggest that networks are, and will continue to be, key drivers of interconnected improvements in comprehensive cancer care at a regional, national, and international level through a “systems-thinking” approach. Networking between CCCs was described across Europe [11,76], Germany [82], USA [78], and India [19], to support and enable government policy, innovative and equitable high-quality research, and improved patient outcomes. Networks of CCCs played a key role in developing best-practice guidelines [78] and patient pathways that can support standardized high-quality care [98]. Research networks between CCCs can enable multi-center, large-scale research to be conducted, such as longitudinal studies, registries, and biobanks, which can lead to breakthroughs for rare cancers and minority/vulnerable populations [25,99]. Descriptions of networked approaches to comprehensive cancer care in low- and middle-income countries were described in the recent literature [94,95].

The literature highlighted health equity and inclusiveness is an opportunity area for CCCs to focus improvement efforts. Inequities surrounding access to care [56] and clinical trials [91] in CCCs in high-income countries were reported in included sources in this review, and substantiated by findings of our recent systematic review [16]. Explicit approaches are needed to combat health equity and demonstrate measurable differences. Governments in the US and Europe have published formalized health equity agendas, describing clear strategies to address health disparities [13,43,44,89,90]. Similarly, a key focus of the new Australian Cancer Plan is improving equitable access and outcomes [100]. In the European Union, networked CCCs are driving Europe’s Beating Cancer Plan with a goal for all members states to have at least one accredited CCC by 2025 as part of the European Network of CCCs [11]. In the US, where CCCs are positioned within the healthcare free market, NCI-designated CCCs must demonstrate corporate social responsibility and citizenship, and attract and retain patients from minority backgrounds equal or higher to their representation in the population [101]. Lengthy, expensive treatments that are far from home may be difficult to afford for people with limited cover from health insurance policies [101]. Local networks where CCCs reach out into the community may help to address this issue. However, national healthcare policy changes are also required in the US to support equitable access to care [101].

A “hub-and-spoke” networked approach to delivery of comprehensive cancer care is an alternative approach for CCCs to achieve their goals and function effectively as important “cogs” in cancer control efforts [9]. For practical reasons, CCCs cannot be ubiquitous geographically and across the cancer trajectory, and most people with cancer will not receive care in a CCC [51]. There are many advantages to receiving care close to home or in a smaller community setting, which is often more accessible and can provide appropriate and high-quality care at lower cost and less inconvenience to patients and families [102]. A networked approach to comprehensive cancer care that positions a CCC as the hub within a geographical region, working with smaller, community health service providers and local health care teams, can serve the individual needs of the community or population and provide equitable access to care. This approach to cancer infrastructure and services can be a viable solution to countries with geographical disparate populations [92] or those lacking the substantial resources that are required to build stand-alone CCCs [94]. In any resource setting, CCCs may be best suited to provide care for certain patients based on clinical need. Notably there were little data to suggest that people with lived experience of cancer are significantly involved in the development of CCCs and their services delivery models; this needs to be rectified to ensure a person-centered approach to comprehensive cancer care. These considerations will be important for future framework development and spatial analysis research informing development of CCCs and their networks to support equitable access to high-quality cancer care.

The findings of this review are slanted towards resource-rich settings where CCCs have historically been developed and maintained. Of note, the development of CCCs is not solely resource-dependent, but also contingent on political commitment, policy alignment, and national cancer strategies. We acknowledge that very few sources were found from low- or middle-income countries, including throughout South America, India, or Asian regions. Subsequently, there is underrepresentation of the characteristics and impact of CCCs in portions of the world largely extending care and services to under-served populations. Comprehensive cancer care delivery in resource-constrained settings may be more likely to rely on a networked approach rather than standalone CCCs, to overcome challenges in scarcity of resources. Such models of comprehensive cancer care may also have innovative approaches to provide equitable access to care for priority and under-served populations. As this review focused on CCC, exploration of alternative models of comprehensive cancer care was beyond the scope of this work. However, we acknowledge the importance of further research to understand and strengthen comprehensive cancer care delivery in resource-limited settings.

### 4.3. Key Recommendations

Based on the findings of this review, we make five key recommendations: (1) focus on all interconnected attributes of CCCs; (2) systems of accountability and governance for CCCs; (3) the need for robust evidence on impact of CCCs; (4) emphasis on networks and networking of CCCs; and (5) continued and increased focus on health equity (Table 7).

### 4.4. Strengths and Limitations

Despite efforts to identify all sources and studies reporting attributes and impacts of CCCs, we were limited to including only those that used the term “CCC”, meaning some that were relevant may have been missed. Only sources and studies in English were included, meaning some relevant information may have been missed (i.e., German literature). The exclusion of websites in gray literature searches may have excluded some leading CCCs. For example, there was a notable absence of sources reporting on UK CCCs, which have played a key role in the establishment of accreditation and designation programs [5,36], a local networked approach to equitable comprehensive cancer services [103], and home to a CCC of Excellence as designated by EACS [104]. Importantly, low-and middle-income countries were not well represented, which influences the application of these findings to a large population of cancer care provider settings; this also demonstrates that the development of CCCs in these countries has been slower compared to high-income countries, possibly due to limited resources and low-priority policy agendas. Despite these limitations, our review provides a methodologically rigorous, thorough, up-to-date, and evidence-based summary and synthesis of the international literature around CCCs.

## 5. Conclusions

Interconnected core attributes and synergistic impacts of CCCs were reported across six themes in mostly opinion-based sources. The results highlight the importance of all attributes and the need for more evidence highlighting the impact of CCCs. The findings also suggest that CCCs are yet to reach their full potential, with anticipated benefits dependent on accountability, effective networking, and focus on health equity at a local, national, and international level. We recommend that countries with well-established, well-resourced comprehensive cancer care networks prioritize equitable partnerships with resource-limited settings. These collaborations should focus on strengthening locally led cancer care, research, innovation, and education to enhance sustainable, high-quality, and accessible cancer services.

## Figures and Tables

**Table 1:** Eligibility criteria.

Inclusion Criteria	Exclusion Criteria
English language Published Jan 2002 to May 2024 Peer reviewed publications—primary research or opinion Unpublished sources—websites, reports, books/chapters, theses Described key attributes (structural or process characteristics) and/or anticipated or realized impacts derived from attributes of a CCC or across multiple CCCs Accreditation and designation programs of CCCs	A single-center experience of accreditation Report on a specific service or practice at a single center (i.e., model of care, technique of biopsy) “News” and marketing pieces reporting individual sites achievement Websites of CCCs (predominantly “public facing” consisting of marketing and “news”) Annual reports of CCCs (reporting on achievements of single CCCs)

**Population:** Not applicable; focus is on comprehensive cancer centers; **Concept:** Comprehensive cancer centers’ characteristics and impacts, and changes in these over time in the literature; **Context:** All settings.

**Table 2:** Summary of included sources in scoping review.

		N	%	Sources
Total number of sources		81	100	
Source type	Peer-reviewed publications opinion, commentary, review (*n* = 26) quantitative observational (*n* = 13), mixed methods or qualitative (*n* = 10)	49	61	[3,4,5,10,13,19,20,21,22,23,24,25,26,27,28,29,30,31,32,33,34,35,36,37,38,39,40,41,42,43,44,45,46,47,48,49,50,51,52,53,54,55,56,85,87,88,89,90,91]
	Book chapters *	18	22	[57,58,59,60,61,62,63,64,65,66,67,68,69,70,71,72,73,74]
	Websites	7	9	[11,75,76,77,78,92,93]
	Policy/framework documents	4	5	[79,80,81]
	White papers	2	2	[82,83]
	Thesis	1	1	[84]
Publication date +	Period I: 2002–2009	4	5	[2,4,24,26]
	Period II: 2010–2019	28	37	[5,19,20,21,22,23,25,26,27,28,29,30,31,32,33,34,35,36,37,38,39,41,46,48,55,74,80,84]
	Period III: 2020–2024	42	56	[3,10,13,40,42,43,44,45,47,49,50,52,53,54,56,57,58,59,60,61,62,63,64,65,66,67,68,69,70,71,72,73,81,82,85,86,87,88,89,90,91]
Country/ region of focus	Europe all of Europe—including UK (*n* = 17) Italy (*n* = 7) Germany (*n* = 6) France (*n* = 1) Eastern Mediterranean region (*n* = 1) Czech Republic (*n* = 1) Europe and USA (*n* = 1)	34	42	[3,4,5,10,11,13,21,22,23,25,27,28,29,30,31,32,33,36,37,40,41,45,46,49,51,76,77,79,81,82,84,85,87,88]
	United States of America	23	28	[20,24,34,35,38,39,42,43,44,47,48,50,52,53,54,55,56,75,78,89,90,91,93]
	International focus * (any country)	19	24	[57,58,59,60,61,62,63,64,65,66,67,68,69,70,71,72,73,74,80]
	Australia	2	3	[83,92]
	Singapore	1	1	[26]
	India (and United Kingdom)	1	1	[19]
	Africa	1	1	[94]
Setting	No specific setting—discussion on CCCs	37	46	[3,4,22,24,25,27,28,32,40,43,44,49,51,57,58,59,60,61,62,63,64,65,66,67,69,70,71,72,73,74,75,76,77,78,79,80,81]
	Focused on CCCs in a region/country (*n* = 14) belonging to a CCC network (*n* = 6) accredited by an organization (*n* = 4)	36	45	[5,10,11,13,19,20,21,23,29,30,33,34,35,36,37,38,39,41,42,45,46,47,48,50,52,55,82,84,85,87,88,91,92,93,94]
	Focused on a single CCC	8	10	[26,31,32,53,56,85,89,90]
Population data derived from	Nonspecific	43	53	[3,4,11,24,25,27,28,29,40,41,49,51,57,58,59,60,61,62,63,64,65,66,67,68,69,70,71,72,73,74,75,76,77,78,79,80,81,82,84,87,88]
	CCCs (multiple or single)	26	32	[5,10,19,20,21,22,23,30,33,34,35,36,37,38,39,42,46,47,50,54,55,92,94]
	Staff within CCCs	5	6	[45,48,53,89,90]
	Patients within CCCs	4	5	[52,56,85,91]
	Attendees at strategic meetings	3	4	[13,43,44]
Cancer type of focus	Cancer in general or various types of cancer	80	99	[3,4,5,10,11,13,19,20,21,22,23,24,25,26,27,28,29,30,31,32,33,34,35,36,37,38,39,40,41,42,43,44,45,46,47,48,49,50,51,53,54,55,56,57,58,59,60,61,62,63,64,65,66,67,68,69,70,71,72,73,74,75,76,77,78,79,80,81,82,83,84,85,87,88,89,90,91,92,93,94]
	Breast cancer	1	1	[52]
Purpose of sources	Provided practical guidance on developing CCCs *	21	17	[25,49,56,88,95]
	Described characteristics, services, or practices of CCCs	17	14	[10,24,26,27,30,39,43,44,50,51,53,74,80,89,90,91,94]
	Described clinical service provision in CCCs (including access to care)	10	8	[20,34,35,38,39,42,45,47,55,85]
	Reported vision and role of CCCs	9	7	[3,4,23,28,41,46,79,81,87]
	Described vision and establishment of networks of CCCs	8	10	[5,19,29,31,32,37,92,93]
	Described rationale, aim, development, implementation and experience of accreditation and designation in CCCs	7	6	[5,21,22,33,36,78,84]
	Described accreditation and designation programs and criteria	6	5	[11,54,75,76,77,82]
	Measured patient or provider impacts associated with CCCs	2	2	[48,52]
	Budget submission	1	1	[83]

+ excluding 7 websites with no publication date * included 17 book chapters within 1 edited book [95].

**Table 3:** Reported attributes and impacts of CCCs, reported under key themes.

Attributes	Setting	Impacts	Setting
Clinical service provision			
Comprehensive, multidisciplinary, and varied clinical services (major and complementary oncology disciplines)—‘one stop shop’ approach [4,5,21,26,29,32,41,51,80,82,95]. Core services include medical imaging, laboratory medicine, pathology, surgery, chemotherapy, radiotherapy, supportive care, palliative care, inpatient care, outpatient care, emergency care, pharmacy [80]. Core infrastructure includes infection prevention and control facilities, appropriate clinical and support services facilities, equipment and technology, health records, hospital registry, human resources, communication resources [80]. All clinical services within the CCC ideally in one visible location, under one roof [4,26] but can be located across various sites [83]. High-quality clinical services, and complex care delivery throughout care pathway (i.e., prevention, diagnostics, treatment, follow-up, end-of-life care) [4,21,26,30,51,95]. Managing quality of life through various support care, integrated and complementary care services [4,20,26,27,30,34,35,38,39,42,95]. Innovative and experimental services (i.e., ovarian cryopreservation, new diagnostic methods, immunotherapies) [20,21,41,83]. Technology-backed care delivery, e Hospital and information systems [31,51]. Alignment of research, care, and education [3]. Protocolised, standardised treatment pathways [35], excellence in patient centered care delivery linked to clinical pathways [21,31,51]. Focus on holistic care [24,42]. Integration of multidisciplinary supportive care and complementary care alongside cancer treatment [34,39]. Consumer engagement in research and service delivery [31,83,84].	Europe, Singapore, International International International Europe, Singapore, Australia Europe, Singapore, International, France USA, Europe, International Europe, Singapore, USA, International USA, Europe Europe, Australia Europe USA, Europe USA USA Europe, Australia	Provide state-of-the-art comprehensive multidisciplinary care of patients throughout clinical pathway [29,31,41]. Critical mass of patients (develops expertise) and access to resources [10,40,49]. Improved patient outcomes and quality of care [53]. Delivery of more complex treatments [29]. Increasing availability of supportive care and complementary care services, and screening for distress [34,39]. Integrated healthcare information systems that improve patient safety, information sharing, patient wait times [31]. Stratification of patients for distinct treatment pathways [40]. Reduced travel time for patients if all services under one roof [53]. High quality cancer care provided across multiple sites and across regional settings due to networks and collaboration [83]. Shift to prevention centered cancer care can improve population health, respond to shortage of healthcare resources (staff and building space), and reduce carbon emissions from cancer care [88].	Europe Europe USA Europe USA Europe Europe USA Australia France
Research, data, and innovation			
Expertise and strength in conducting ‘bench to bedside’ research—spanning basic science to translational research [3,4,5,10,23,26,40,41,50,80,82,95]. Research focused on: innovative precision medicine [23]. establishment of biobanks due to privileged access to biospecimens and longitudinal data [25,36]. cancer prevention strategies [28]. pioneering innovation, and new therapeutic pathways [3]. longitudinal research [40]. cost-effective, home-grown innovations [84]. Dedicated clinical trials infrastructure and large numbers of diverse patient groups [31,51,95]. Uniform quality standards for research [19]. Clinically relevant research due to clinician researchers—with multidisciplinary academic expertise [28]. Protected research time for clinician researchers [49]. State-of-the-art technology and infrastructure for research [40]. Sharing resources, data, and research infrastructure with other organisations to support research [23,46,49,84]. Conduct clinical trials across networks to support less experienced CCCs [19,23]. Consumer engagement in research [31,84]. Information and education for patients and families [41]. Formal mechanisms to gather community input regularly [44]. Partnerships with community-based health care organisations [44].	Europe, Singapore, USA Europe Europe, UK Europe Europe Europe Europe, UK Europe, International India, UK Europe Europe Europ Europe, UK India, UK, Europe Europe Europe USA USA	High patient accrual in clinical trials and other research [10,36,41,49,51]. Greater research activity compared to non CCCs [10]. Increased clinical effectiveness/efficiencies and reduced costs [51]. Translation of research from basic science to clinical implementation—and into clinical guidelines and practice [28,40,45,51,81]. Higher academic output of staff at CCCs compared to non-CCCs [10,48,51]. Networks of CCCs: ○ strategically address key relevant areas of cancer research [46]. ○ facilitate innovative clinical trials through international collaborations [13,33]. ○ collaborate to increase focus on rare cancers [33]. ○ develop specific advanced projects in research [33]. ○ lead to greater availability of clinical trials to patients all over the country [10,28,33]. ○ lead to greater number of eligible patients for clinical trials [33,40,49]. ○ improve local research capacity in middle-income countries [19]. collaborate to increase focus on rare cancers [33]. Establishment of cancer registries [31]. Patents, spin-off companies [51]. Innovative research and major breakthroughs stemming from biobanks [25]. Integration of multidisciplinary care and research [28]. Sharing of expertise between researchers and clinicians [28]. High levels of patient experience and satisfaction [53]. Streamlined patient pathways [44]. Meeting patients’ information-seeking needs around complementary therapy [39]. Increased patient empowerment through consumer engagement in all aspects of service delivery [23,33]. Strengthening community outreach capacity and support for delivery of high-quality care in community by working with community leaders [44].	Europe, UK, USA Europe Europe Europe USA, Europe Europe Europe Europe Europe Europe Europe India, UK Europe Europe Europe Europe Europe Europe USA USA USA Europe USA
Education and clinical support			
Comprehensive, interdisciplinary, high-quality education, training and mentoring of healthcare professionals and all staff within organisation—in clinical care and research [10,19,25,36,41,74,80,81,95]. Availability of standard operating procedures and best practice guidelines for staff [41]. Research, care and education are aligned [3]. Delivery of post graduate cancer programs and medical training programs [26,83]. Education, training, and mentoring of healthcare professionals outside of organisation—i.e., tumor boards, online resources, conferences [41,47,83]. Public cancer education [26].	Europe, USA, UK/India, International, Europe Europe Europe, USA, Australia Singapore	High levels of staff satisfaction [24,53]. Greater teaching and collaboration outcomes when staff located in one building [53]. Support national health plans by upskilling staff [25]. Information exchange regarding provisions of complex clinical care [29]. Career advancement opportunities for staff [81]. Completion of post graduate studies by staff [83].	USA USA Europe Europe Europe Australia
Leadership and networks			
Structured, supportive, and committed leadership and management required within CCCs [10,21,36,53,80]. Provide ‘second opinions’ to people diagnosed outside of a CCC [52,85]. Networks of geographically dispersed CCCs across a nation or continent: focused on common aims such as improving clinical care, equity, access to care, and patient outcomes—and strengthening research, with a view to international collaborations [3,11,19,33,37,78,80]. influence decision-makers on cancer related issues, inform and integrate with national cancer strategies [3,4,11,49,51]. act as focal points for national cancer control programs in low- and middle-income countries [74]. involved in development of guidelines, clinical pathways and provide technical and organisational support to national patient services [11,25]. involved in primary prevention, early detection, and screening programs [80]. network of CCC in middle-income country, supported by alliance in high-income country [19]. Networks between a CCC and community providers and smaller/non-specialised hospitals: Outreach and support for community providers and with smaller hospitals across catchment areas, and support for community oncologists in delivery of best practice care [41,47,51,79,80]. Networks and alliances can strategically share management structures and resources [11,23,29,40]. Alliance models of CCCs—networked services rather than all services under one roof [83,92,94].	International, USA, Europe USA, Germany Europe, USA, International Europe International Europe, USA International India, UK Europe, USA, International Europe Africa, Australia	Networks of CCCs: attract world-renowned experts and enhances collaboration [1,76]. support guidelines development, engagement with stakeholders, promotion of effective policies [1,11]. enhance research capacity across care continuum [3,11,19,22,37]. train next generation of staff [22]. form linkages with cancer patient organisations [33]. Alliance models of CCCs (networked services) can support geographically dispersed cancer services, enabling people with cancer to receive care close to home [83,92]. Leadership of CCCs: Can reduce cancer related mortality and morbidity nationally or internationally [4,11]. Support national cancer control systems, effective healthcare systems, improvement in health of population [11,74,95]. All services in one location increases engagement in adaptive behaviour of staff and enactment of organisational citizenship behaviour [53]. Clinical support for regional areas and community hospitals [52]. Clear vision and articulated intention [36].	USA, Europe USA, Europe India, UK, Europe Europe Europe Australia Europe International USA USA Europe, UK
Health equity and inclusiveness			
Availability of one or more specialised cancer center in every country—where possible a CCC [11,74,79]. Networks of CCCs focused on health equity in research and clinical care provision [3,11,82]. Commitment to outreach and engagement with smaller hospitals and networking with community across catchment areas to address equity [3,51,54,79,82]. Adopting and resourcing an explicit health equity approach [43]. ○ using local data to understand disparities [44]. ○ understanding and addressing structural barriers to health equity [43]. ○ advancing relevant health equity policies [43]. Self-help and patient advocacy groups for patients and families [41]. Challenges regarding equitable access to timely care at CCCs for vulnerable populations [55,56]. Global program—developed strategic global initiatives to ensure awareness, accessibility, and relevancy of NCCN resources [93].	International Europe USA, Europe Europe, USA, International USA USA USA Europe USA	Translational research focused on health equity [50]. Reduced inequalities in diagnosis, treatment, care, and access to clinical trials in metro and regional areas [51,54,83]. Increased quality of care and clinical trial enrolment of minority populations [44]. Networks of CCCs: ○ increase availability of clinical trials to patients across country [33]. ○ facilitate adequate numbers of patients for innovative personalised/precision cancer medicine [3,28]. Increase focus on rare types of cancer through collaboration in networks [33]. Increased ability to secure grant funding if research focused on equity of local catchment area [54]. Collaboration between high- and, and low- and middle-income countries can increase global access to high-quality, high value cancer care [93].	USA Europe, Australia, USA USA Europe Europe Europe USA USA
Accountability and governance			
National bodies and organisations that oversee CCCs, networks of CCCs, and accreditation and designation programs [4,11,29,46,78]. CCCs underpinned by quality standards and national or international accreditation and designation programs [1,11,32,34,49,76,77,81,82,84,95]. CCCs have sophisticated organizational structures and governance and resourced with large budgets [10,49,80]. Accreditation criteria clearly describes the essential components and prescribed standards, distinguishing CCCs from other types of cancer centers [1,11,76,77,82].	Europe, USA Europe, USA, International, UK Europe, International Europe, USA	Accreditation and designation programs: ○ define and advance high-quality patient-centered cancer care [1,11,76]. ○ contributes to consistent procedures for multidisciplinary teams [11,76]. ○ improve risk management systems [76]. ○ enables allocation of funding [36,46]. ○ improves integration of research into clinical care, and efficient use of resources [76]. ○ enhances education and training of staff [81]. ○ facilitate the creation of international scientific advisory boards [76]. ○ supports consumer engagement in research and service delivery [76].	Europe/USA Europe Europe Europe Europe Europe Europe Europe

Note: Attributes were defined as structural or process characteristics, and impacts were defined as anticipated or realized impacts derived from attributes, relating to comprehensive cancer centers.

**Table 4:** Characteristics of current CCC accreditation and designation programs.

National Cancer Institute (NCI) Accreditation and Designation Program—USA (est. 1973)	German Cancer Society (Deutsche Krebsgesellescaft) Cancer Center Certification Program—Germany (est. 2007)	Organization of Europe Cancer Institute (OECI) Accreditation and Designation Program—Europe (est. 2008)
**Summary:** Accredited cancer centers that “meet rigorous standards for transdisciplinary, state-of-the-art research focused on developing new and better approaches to preventing, diagnosing, and treating cancer”. Three types of designation: (1) comprehensive cancer center; (2) clinical cancer center; and (3) basic laboratory cancer center. CCCs network across USA in National Comprehensive Cancer Network. **Criteria**: Core features of NCI-CC: (1) policy of inclusion; (2) excellence in cancer research; and (3) education and dissemination. Essential characteristics of an NCI-CC: Facilities/physical space Organizational capabilities Transdisciplinary collaboration and co-ordination Cancer focus Institutional commitment Center director NCI designated CCCs must meet NCI standards as CC in cancer prevention, clinical services, and research. In addition, CCCs must demonstrate an added depth and breadth of research and substantial transdisciplinary research that bridges the relevant scientific areas.	**Summary:** Cancer centers which form a network of qualified and jointly certified multi- and interdisciplinary, cross-sectoral, and where applicable cross-regional sites, which provide the complete care for people with cancer. Three tier model of designation: (1) comprehensive cancer center (oncology center of excellence); (2) oncology center; and (3) organ cancer center. **Criteria:** Oncology centers are certified to provide multi-disciplinary, state-of-the-art treatment for a range of specific cancer types. CCCs must be a certified oncology center and must fulfill additional requirements for research and education. CCC is a leading oncology center with major research aims and specifically care for rare cancer diseases and special issues. In addition, CCC must: Provide best-practice, evidence-based care for patients with cancer types not covered in oncology centers. Act as focal points of a regional care network and drive innovative developments in region. Demonstrate reasonable depth and breadth in research including basic laboratory, clinical and preventative, cancer control and population-based work.	**Summary**: International accreditation program based upon standards for high-quality cancer care, research, education, and patient centeredness, with the aim of enhancing collaboration in European cancer centers. Three types of designation: (1) comprehensive cancer center; (2) cancer center; and (3) comprehensive cancer network. Cancer Core Europe—research network of leading European CCCs. **Criteria**: All OECI designated centers must have: An identifiable organizational entity with a clear governance A direct provision of an extensive range of high-quality cancer diagnostics and care tailored to the individual patient’s needs. A culture of learning and improving the professional and organizational quality of care. In addition, CCCs are required to demonstrate: A high level of infrastructure, expertise, and innovation in cancer research, especially in translational and clinical research, but also in many cases including basic science. Either strong University and Research Institute links, or a university partnership as part of the Comprehensive Cancer Center Extensive international networking

At the time of publication, the European Network of Comprehensive Cancer Centers accreditation and designation program was under development.

**Table 5:** Results of primary research.

Author	Study Design/Aim	Sample	Key Findings
Quantitative studies *n* = 13			
Hammer 2015, USA [34]	Survey—to provide an updated assessment regarding supportive care services and subjective effectiveness of such services (comparing changes that have occurred over a 17-year period).	NCI-CCCs and NCI-CCs (*n* = 31/41, 76% response rate).	From 1994 to 2011, integration of supportive care services, availability of complementary services, and the number of pain and palliative care services offered increased. There was also an increase in patient and family advisory council and distress screening. Gaps remained in end-of-life care and hospice services. Pain management was offered by staff in all centers, followed by nutritional counselling (88%), a palliative care clinic (88%), ostomy care (76%), and rehabilitation (72%). Genetic counselling was available at 81% of institutions. The most offered social services were navigation (96%) and advocacy (92%). The following complementary services were offered by staff by 84% of the institutions surveyed: relaxation/meditation, guided imagery, art therapy, family/caregiver programs, and bereavement.
Platek 2015, USA [35]	Survey—to determine the prevalence and types of outpatient clinical nutrition services available at NCI-CCCs.	Telephone survey at NCI-CCCs *n* = 32/40 (80% response rate) with registered dieticians.	Most (94%) CCCs had referral or consultative based services for outpatients with a nutrition profession such as a registered dietician (not consistently part of the outpatient multidisciplinary team). Three quarters (73%) of the CCCs monitored outpatients regularly, but only half (48%) followed a clinical nutrition protocol. Specific nutritional services were provided at 56% and 46% of CCCs for head and neck and gastrointestinal cancers, respectively. For those centers that provide clinical nutrition services via referral or consult system (*n* = 30), 23 said that they followed referred patients regularly. Eleven of these used an evidence-based protocol; 91% of the 11 stated that the protocol was part of standard of care. Sixteen of these respondents reported that clinical nutrition services offered to head and neck patients were referral or consult based, and 15 centers provided head and neck patients who were referred with regular follow-up. Of these 15, seven followed a specific evidence-based protocol, and six out of the seven incorporated these protocols into standard of care.
Yun 2017, USA [39]	Website review—to determine the growth of integrative medicine in leading academic cancer centers in the USA as reflected by their public-facing websites.	NCI-CCCs (*n* = 45)	Between 2009 and 2016, NCI-designated CCCs increasingly present integrative medicine content on their websites, and most of them provide these services to patients in the same health systems. Compared with the results from 2009, the number of CCCs providing information on integrative medicine increased for all modalities except guided imagery. On the 45 NCI-CCC websites, the most common integrative medicine therapies mentioned were exercise (97.8%), acupuncture and meditation (88.9% each), yoga (86.7%), massage (84.4%), and music therapy (82.2%). Most websites provided information on nutrition (95.6%), dietary supplements (93.3%), and herbs (88.9%). The most common therapies offered in CCCs were acupuncture/massage (73.3% each), meditation/yoga (68.9% each), and consultations about nutrition (91.1%), dietary supplements (84.4%), and herbs (66.7%).
Rolland 2018, USA [38]	Website analysis—to understand the types of posttreatment survivor-specific resources available on CCCs’ websites.	Websites of NCI-CCCs (*n* = 47).	Although 75% (*n* = 35) of CCCs had some information on their websites, limited survivor-specific services information was available for patients, caregivers, or clinicians. 45% (*n* = 15) CCCs websites had explicit information on surveillance; 36% (*n* = 17%) discussed prevention activities; 43% had information regarding survivor-specific mental health; 40% had any reference to survivorship cancer plans; and 51% offered information about a general survivorship program. NCI-CCCs serve as a model to community oncologists and clinics in the delivery of best-practice survivorship care. CCCS are expected to be leaders for community providers, and function as sources of information for survivors, caregiver, and clinicians in navigating care after treatment.
Kim 2019, USA [48]	Analysis of academic output—to examine the influence of Surgical Society Oncology membership with NCI status on the academic output of surgical faculty at NCI-CCCs and NCI-CCs.	Surgeons (*n* = 4015) at top 50-ranked university based and top 5-ranked hospital-based NIH funded departments for surgery (*n* = 29 NCI-CCCs, *n* = 12 NCI-CCs, *n* = 13 non-NCI centers).	Surgeons at NCI-CCCs had significantly higher academic output and NIH funding. NCI-CCC designation and Surgical Society Oncology membership had synergistically effect on increased citations and citations. At CCCs, 22.7% of surgical faculty had a history of or current NIH funding, compared with 15.8% at the CC and 11.8% at the non-NCI centers. CC surgical faculties were better funded by NIH R01/P01/U01 grants (9.5%) compared with those from NCI CC (7.9%) and non-NCI center s (6.8%). CCC (11%) and non-NCI (8.5%) faculty were more likely to have SSO membership than at NCICC (4.6%), *p* < 0.05. CCCs were more likely to have surgical faculty in leader-ship positions (13.7%) compared with NCICC (7.9%) and non-NCI centers (10%), *p* < 0.05. Although CCC had a trend for more surgical faculty with PhDs or MD-PhDs (12%) versus those at NCICC (6.5%) and non-NCI centers (9.9%), this did not reach statistical significance. NCI-designated comprehensive cancer centers demonstrate research excellence at every academic level. The median publications and citations *p* < 0.05
Gahr 2020, Germany [45]	Survey—to evaluate the implementation of best-practice recommendations for the integration of palliative care in CCCs.	Director of OECI-CCCs (*n* = 15/15, 100% response rate) in Germany.	All CCCs (*n* = 15) had a palliative care unit. 13/15 units had palliative care specialists available 24 h a day. 11/15 CCCs offered specialist palliative care within inpatient oncology departments. 9/15 CCCs had at team of at least 3 multidisciplinary clinicians (medical, nursing, allied health). 12/15 CCCs had facilities for specialist palliative care in oncology outpatients. 11/15 had outpatient palliative care clinics. All CCCs had specialist palliative home care available. 11/15 CCCs enquire about living wills and power of attorney on admission. 9/15 CCCs submit data to a National Hospice and Palliative Care Registry. 6/15 CCCS had a quality concept for managing patients at the end of life (i.e., pathways). 12/15 CCCs had palliative medicine integrated into research structures of CCC. 10/15 CCCs have a structural concept to support research and teaching in the field of palliative medicine. 5/15 CCCs had European Society Medical Oncology certification. The majority of the German CCCs already fulfilled essential organizational and structural requirements of the Palliative Medicine Working Group guidelines. Variation existed around availability of various palliative care services.
Desai 2021, USA [42]	Systematic review of websites—to compare the availability of integrative medicine therapies in NCI-designated CCCs, and community hospitals.	NCI-CCCs (*n* = 51) and community hospitals (*n* = 100).	Community hospitals offered fewer integrative medicine therapies as compared with CCCs. Availability of acupuncture (56% vs. 76.5%, *p* = 0.01), meditation (63% vs. 82.4%, *p* = 0.02), and music therapy (55% vs. 74.5%, *p* = 0.02) was significantly lower at community hospitals compared with CCCs. For massage (80% vs. 84.3%, *p* = 0.52), yoga (79% vs. 84.3%, *p* = 0.43), fitness (72.6% vs. 85%, *p* = 0.07), and Tai Chi (45% vs. 51%, *p* = 0.49), there was no significant difference between community hospitals and CCCs Integrative care was significantly lower in community hospitals serving lower-income populations. Equitable access to evidence-based integrative medicine in community hospitals is needed.
Kehrloesser 2021 Europe [10]	Secondary analysis—to identify the hallmarks common to all cancer centers and the distinctive features of CCCs using OECI accreditation data.	OECI-CCCs and CCs (*n* = 40) in 18 European countries.	Compared to CCs, CCCs: Had better overall compliance with OECI quality standards—with the main difference in leadership and management, and research, innovation, and development. Had better organizational structure and governance—specifically around corporate strategic planning, quality of patient outcome data, diagnostic trends reported by centers, evaluation the effect of improvement actions. No difference in areas of cancer prevention measures, cancer treatment and care standards, teaching and continuing education, and patient centeredness. Provided care for double the number of oncology patients. Managed larger budget for oncology care (median EUR 150.1 M vs. EUR 68.4 M). Higher number of total peer-reviewed national and international publications per year (median 370 vs. 104). Significantly more clinical trials open to recruitment in CCCs (median 162 vs. 42). Higher patient numbers recruited to prospective interventional trials per index year (median 894 vs. 123), Had higher volume, quality, and integration of translational research (i.e., high-impact publications, and clinical trial activity. Were significantly stronger than CCs in research collaborations, organization of clinical research, processes of intellectual property and innovation, and infrastructure for biobanking; and Were also more consistent in having a robust scientific knowledge transfer program, being subject to regular external review, and in engaging an international Scientific Advisory Board.
Mueller 2021, USA [50]	Review of NCI administrative data—to summarize the characteristics of NCI-funded dissemination and implementation grants in CCCs and CCs to understand the nature, extent, and opportunity for this type of translational work.	NCI-CCCs (*n* = 51), NCI-CCs (*n* = 13), and active affiliates.	62% of CCCs (*n* = 32/51) and 38% of CCs (*n* = 5/13) held a dissemination or implementation grant. Half of the grants focused on specifical cancers, most commonly colorectal, breast and cervical. Grants that were not focused on specific cancer focused more generally on health behavior, community outreach, or health information technology. Almost two thirds of the grants focused on health equity. The most common health equity topics were: (1) social, economic, or structural determinants of health; (2) race or ethnicity; (3) social needs; 4) socioeconomic status or income; and (4) rurality. There is considerable room for development to support the NCI’s mission to support translation of research.
Kalra 2022, USA [47]	Case study—to describe an oncologist-only question and answer (Q&A) website (Mednet) that aimed to document insights from Tumor Boards to provide educational benefits to the oncology community.	Website hosted by 16 NCI-CCCs.	The Mednet was developed in 2014 as a physician-only online platform with a mission to facilitate knowledge sharing from academic to community physicians for patients to get high-quality care despite where they are treated. The platform was designed for community oncologists to ask non–case-based clinical questions from experts and for the expert answers to be part of a large and searchable Q&A database that would be accessible at any time to physicians with similar questions. Between Dec 2016 and Jul 2021, 534 answers to 368 questions were posted from 16 NCI-CCC sites. Answers came from 123 academic physicians and were peer reviewed by 93 academic physicians. Q&As were viewed 147,661 times by oncologists at 3515 institutions from all the 50 states of the USA, including 5131 community oncologists. Of the 1063 responses to a survey on how the Q&As affected clinicians’ practice, 646 (61%) reported that it confirmed their current practice, 163 (20%) indicated that a Q&A would change their future practice, 214 (15%) reported learning something new, 20 (2%) indicated that their practice differs, and 20 (2%) chose “other” as their response.
Kirtane 2022, USA [56]	Retrospective chart review—to examine the timing of patients’ presentation at an NCI-CCC relative to their diagnosis and demographic characteristics.	Patients with breast, colon, lung, melanoma, and prostate cancer who presented to a single NCI-CCC between 2008–2020	African American patients had a longer time between diagnosis and presentation to the NCI-CCC compared to White patients (median 510 vs. 368 days). African American patients were also more likely to have received their initial cancer care outside of the NCI-CCC compared to White patients (odds ratio 1.45, 95% confidence interval 1.32–1.60). Furthermore, Hispanics were more likely to present to the NCI-CCC at an advanced stage compared to non-Hispanic patients (Odds ratio 1.29, 95% 1.05–1.55).
Alaniz 2023, USA [54]	Online survey exploring the impact of Community Outreach and Engagement component has on the overall Cancer Center Support Grant merit descriptors and score for NCI-CCCs and clinical centers.	NCI-CCCs and clinical centers across USA N = 48/62 (77% response rates).	Community Outreach and Engagement component merit descriptors are strongly correlated (Spearman’s rank correlation coefficient r = 0.544, *p* = 0.0003) with Cancer Center Support Grant scopes for CCCs (but not clinical centers). CCCs that score better in Cancer Center Support Grant applications may receive more funding or be eligible for extended renewal cycles. This indicated that Community and Outreach Engagement initiatives are an important investment for NCI-CCCs.
Schulmeyer 2024, Europe [85]	Review of medical records—to determine if first opinions at non-CCCs were guideline concordant, in a cohort of people with cancer seeking second opinions at a CCC in Germany	People with urological, gynecologically, gastroenterological cancers, and sarcomas (2014–2020) who were seeking a second opinion regarding cancer therapy at a CCC (N = 584)	First opinions in non-CCCs were accordance with the guidelines for 54.5% of patients. The median time taken to form a second opinion was 225 min, and the cancer information service was contacted by patients an average of eight times. Obtaining a second opinion at a CCC gives patients an opportunity to receive a guideline-compliant treatment recommendation and enables them to benefit from newer, individualized therapeutic approaches in clinical trials. Establishing patient-initiated second opinions via central contact points appears to be a feasible option for improving guideline compliance.
Unger 2024, USA [91]	Secondary analysis of accreditation data—to identify a contemporary estimate of enrolment to cancer treatment trials across a diverse set of clinical care facilities in the USA.	Accreditation data from 1200 Commission on Cancer programs (2016–2018), representing 70% of all cancer cases diagnosed in USA each year.	Participation in cancer studies (including treatment trials, biorepositories, diagnostic trials, economic studies, genetic studies, quality of life studies, and registry studies) was significantly higher at CCCs compared to non-CCCs (e.g., academic comprehensive cancer programs, community cancer programs, and integrated network cancer programs). Treatment trial enrollment was 21.6% at NCI-designated comprehensive cancer centers, 5.4% at academic (non–NCI-designated) comprehensive cancer programs, 5.7% at integrated network cancer programs, and 4.1% at community programs. One in five patients (21.9%) participated in one or more cancer clinical research studies.
Qualitative or mixed-methods studies *n* = 8 *			
Saghatchian 2014, Europe [5]	Secondary analysis of accreditation data—to describe the landscape of the first 10 participating cancer centers in the OECI accreditation and designation program, and describe their compliance with the standards of the OECI program.	First 10 European cancer centers (*n* = 10) participating in OECI accreditation and designation program (2 academic institutions, 7 public/non-profit, and 1 private).	All 10 cancer centers applied for CCC designation; 5 were designated as CCCs, and 4 as clinical CCs (1 center was awaiting designation at the time of publication, pending major changes). For 5 centers that failed to receive CCC designation 3 had research shortcomings, 1 had research and care issues, and 1 was related to care alone. Criteria related to research shortcomings included lack of publications in journals with high impact factor lack of clinical trials, lack of integration of research into care or between laboratories. Care shortcomings mainly concerned lack of harmonization between patients and quality policy and guidelines. Lack of an identifiable dedicated integrated structure for cancer management was also a key issue in obtaining CCC designation, particularly in cancer centers in large university hospitals.
Berendt 2016, Germany [30]	Delphi—to develop consensus-based best-practice recommendations for the integration of palliative care in German CCCs.	Experts (*n* = 55) from CCCs designated by German Cancer Aid (*n* = 15).	Palliative care (general and specialized) is an integral part of comprehensive cancer care. CCCs are recommended to have an inpatient palliative care consultation service and an outpatient palliative care clinic. The development of multi-professional palliative care consultation teams, outpatient clinics, and the integration of specialized palliative care in consultation hours of other departments and research projects of CCCs are future goals for CCCs in Germany.
Rajan 2016, Europe [36]	Pilot—to test a newly developed Excellence Designation System in translational research in CCCs.	Three OECI-CCCs.	Of the 3 CCCs that applied for the designation of excellence in translational research in the pilot, two were determined to be “excellent” and one “actual potential for excellence”. Key limitations in the CCC that did not achieve excellence were related to biobanking practices, resourcing for novel collaborations, relationships with university regarding discovery, combination drug testing, availability of academic trials. Clinicians from the 3 participating CCCs felt the criteria and process for assessing excellence in translational research was useful for identifying weakness and strengths and could drive improvements in their facility. It was reported that the assessment system should not be burdensome to complete (from a paperwork perspective).
Clayman 2013, USA [20]	Semi-structured interviews—to determine what fertility preservation resources are available in CCCs and how well those are integrated into patient care.	NCI-CCCs (*n* = 30/39, 77% response rate).	CCCs vary widely in implementing fertility preservation-recommended practice to patients. Most sites either had some fertility services on-site or had referral programs. Some hospitals had experimental services, such as ovarian tissue cryopreservation. Few sites had staff with time dedicated to fertility preservation or institutional policies regarding consistent provision of fertility information. CCS are well-positioned to provide an excellent standard of onco-fertility care, but most need to better integrate fertility preservation referral and information into practice. CCCs are resource-laden compared with many community clinics
Hamlyn 2016, USA [55]	Mystery shopper method—to quantify and qualitatively explore variation in accessibility of services and the quality of information provided at NCI-CCCs	NCI-CCCs (*n* = 40/40, 100% response rate).	There was no statistically significant variation between appointment availability for people with private insurance versus Medicaid insurance. Callers who reported having Medicaid insurance had longer wait times (12.7% vs. 7.7% waiting more than 2 weeks) until first appointment compared to privately insured callers. Callers who reported having Medicaid insurance reported differences in experience in qualitative data; “Our cancer center does not] generally take patients with Medicaid unless [it is a] rare cancer that is being studied” and “We don’t take Medicaid HMO [health maintenance organization], so call back when you know [your mother’s] insurance.”
Pasick 2020, USA [52]	Ethnographic method—to explore the feasibility and benefit of second opinions from breast oncologists within NCI-CCCs for African Americans treated at community hospitals.	African American women with breast cancer (*n* = 14).	In “second option” consultations, CCC clinicians offered important recommendations including changing or modifying treatment plans and/or improving management of side effects. All second opinion recommendations were followed by treating clinicians at non-CCC hospitals. Second opinions from oncologists at CCCs is feasible and can improve treatment quality.
Majumdar 2022, USA [53]	Semi-structured interviews and survey—to identify a possible model to explain how merging teams and professions into a unified NCI-CCC might influence healthcare team processes and experience, and patient experience.	Survey: health care professionals employed at CCC (*n* = 20/42, 48% response rate) including medical, nursing, allied health, and administrative staff. Semi-structured interviews: patients receiving outpatient cancer treatment in the hospital (*n* = 50/50, 100% response rate), *n* = 26, 52% male participants).	A range of individual, team, and organizational-level inputs, processes, and outputs were reported to impact on team processes within a newly merged CCC, in turn contributing to critical outcomes such as healthcare professional and patient experience. Reported benefits of the merged CCC included: (1) improved quality of patient care; (2) increased patient satisfaction); (3) satisfied employees); (4) staff learning from learning from/with each other; (5) engagement in adaptive behavior by staff; (6) enactment of organizational citizenship behavior; and (7) having a one-stop shop for patients (and employees who provided ancillary services), with reduced travel time between clinics/units.
Odedina 2024, USA [89]	Impact and logic models—to describe the development of two guide models that address health disparities and reduce cancer burden in local catchment area	Community Advisory Board—3 sites (8–10 members at each site) comprising of survivors, lay caregivers, local cancer advocates, national/regional representatives.	An impact and logic model was developed to serve as a roadmap to monitor progress towards short- and long-term community outreach and engagement goals of the CCC. The community outreach and engagement operational strategies draw upon bidirectional partnership, evidence-based practices, and research facilitation to respond to the critique in the Cancer Center Support Grant application (address cancer health disparities and reduce cancer burden in catchment area). Targeted strategies to engage with the community can help address cancer burden, promote health equity, and eliminate cancer disparities in the CCC catchment area.
Trapl 2024, USA [90]	Semi-structured interviews, national survey, and development and utilization of framework—to examine the experiences and perspectives of community engagement by members of a CCC and create and implement a framework to meet the needs of the entire CCC.	Semi-structured interviews: researchers in the CCC (*n* = 12 interviews) Survey: members of the CCC members (*n* = 86)	Importance of community engagement, and opportunities for bidirectional engagement recognized by members of the CCC. Members of CCC were open to learning new skills, changing approaches, and utilizing services to facilitate engagement and overcome barriers including communication issues, limited awareness of opportunities, and competing priorities.

* Soo 2008 [26] published a case study describing the process of establishing the National Cancer Center in Singapore but did not present any data and is therefore not reported on in this table. Abbreviations: CCC—comprehensive cancer center; NCI—National Cancer Institute; OECI—Organization of European Cancer Institutes; *p*—statistical significance; vs.—versus.

**Table 6:** Summary of changes in the CCC literature across time, according to themes.

Theme	Period I: 2002–2009 (*n* = 4)	Period II: 2010–2019 (*n* = 28)	Period III: 2020–2024 (*n* = 42)
Clinical service provision	Establishment of CCCs [26] Call for CCCs to provide holistic care [24]	Availability of clinical services in CCCs [20,25,34,35] Provision of supportive and integrative care in CCCs [27,34,38,39] Recommendations for care delivery in CCCs [80]	Priority areas for CCCs in treatment and care [83] Guidance on of clinical services in CCCs [95] Evaluation of best-practice recommendations in CCCs [45] Exploration of benefits of second opinions in CCCs [52,85] Availability of integrative medicine in CCCs [42] Description of cancer prevention services led by CCCs [88]
Research, data, and innovation	Nil	Opportunities areas for research in CCCs [28] Development of criteria for excellence in translational research [36,84] Development of research networks of CCCs [23,46] Superior academic output in CCCs [48]	Priority areas for CCCs in research [49,83] Grant schemes for CCCs (general, translational, and community engagement research) [50,54,82] Guidance on clinical trials, research, and translation (staff, infrastructure, processes) in CCCs [40,95]
Education and clinical support	Nil	Development of best-practice guidelines in CCCs [30]	Guidance on education and training, and workforce issues in CCCs [95] Description of oncology question and answer websites led by CCC [47]
Networks and leadership	Strategies for CCCs to align with cancer control programs [79]	Growth and development of networks between CCCs [22,33,37] Research networks between high- and middle-income country [19] Outreach with community stakeholders [41]	Vision for CCC policies and initiatives that will lead to improved quality of cancer care across Europe [81] Networks identified a key priority action for CCCs to support national cancer control plans [3] Guidance on merging of services into a single CCC [53]
Health equity and inclusiveness	Nil	Identification of barriers in equitable access to care at CCCs [55]	Equitable access to care [56] and clinical trials [91] in CCCs Setting health equity agenda, developing strategies and models to address health disparities, and increased focus on community engagement in CCCs [13,43,44,89,90] Role of CCCs in making anti-cancer treatments more affordable [87] CCCs in countries with limited resources [94,95]
Accountability and governance	Description of establishment of CCCs and accreditation and designation programs [4]	Growth and development of accreditation and designation programs [5,21,29,31,32] Structures and processes required for excellence in patient care [51]	Guidance on quality measures in CCCs [95] Description of key features of CCCs as per accreditation data [10]

**Table 7:** Key recommendations based on review findings.

1. Focus on all interconnected attributes of CCCs	Recognition that all key attributes are important and needed for CCCs to reach their full potential. Development of greater understanding around the interconnectedness of attributes, to maximize synergistic benefits.
2. Systems of accountability and governance for CCCs	Systems of accountability and governance are present for all CCCs and networks of CCCs. Key attributes of CCCs are defined, and assessed to optimize accountability, ensure quality, and enabler networking between CCCs. Defining the role of CCCs within a wider system. Sharing of knowledge and experiences is recommended between CCCs with established accreditation and designation programs and those yet to establish systems of accountability.
3. Robust evidence needed on impact of CCCs	Assess if the goals of CCCs are being achieved. Develop a framework to assess the impact of investment in CCCs. Explore impacts of CCCs at a societal, organizational, provider, and patient level. Focus research efforts to address health equity and inclusiveness of priority populations.
4. Emphasis on networks and networking of CCCs	Networks of CCCs to be developed and/or strengthened nationally and internationally to support research and innovation, development of best-practice guidelines, share resources, address health equity and inclusiveness, and influence and support for cancer plans. Networks of Comprehensive Cancer Infrastructures within and across resource constrained settings to enhance quality of equitable care and research. For networks between CCCs and local regions to provide support to clinicians, disseminate evidence and guidelines, introduce new technologies, and target local health equity concerns—driven by local data and community engagement. Networking between CCCs and a local region/community to be established and/or strengthened to provide support to clinicians, disseminate evidence and guidelines, introduce new technologies, and target local health equity concerns—driven by local data and community engagement.
5. Continued and increased focus on health equity	CCCs to address health equity at an upstream level through networks and leadership—influencing governments on cancer control policies. CCCs and networks of CCCs to articulate and support a dedicated health equity and inclusiveness agenda. Work towards equitable access to care and representation on clinical trials of minority groups at CCCs. High-income countries can partner with low- and middle-income countries to support local provision of high-quality care, research and innovation, education and clinical support, and development of programs that provide accountability and governance.

## Data Availability

No new data were created or analyzed in this study. Data sharing is not applicable to this article.

## Supplementary Materials

The following supporting information can be downloaded at: https://www.mdpi.com/article/10.3390/cancers17061023/s1, Supplementary File S1. PRISMA checklist, Supplementary File S2. Database search strategies, Supplementary File S3. Condensed data extraction table, Supplementary File S4. Narrative summary of key attributes and impacts of CCCs, Supplementary File S5. Current CCC accreditation and designation programs.

## Abbreviations

The following abbreviations are used in this manuscript:

CCC	Comprehensive cancer center
NCI	National Cancer Institute
OECI	Organization for European Cancer Institutes
WHO	World Health Organization
