# Supplementary material for: Describing the Core Attributes and Impact of Comprehensive Cancer Centers Internationally: A Chronological Scoping Review
Source: Cancers (Basel). 2025 Mar 18;17(6):1023. doi: 10.3390/cancers17061023 (PMC11940407; doi:10.3390/cancers17061023)
Supplement: Supplementary file 1 [file cancers-17-01023-s001.zip › cancers-3499625-supplementary.pdf]

Supplementary Files

Supplementary File S1. PRISMA checklist

Preferred Reporting Items for Systematic reviews and Meta-Analyses extension for Scoping Reviews (PRISMA-ScR) Checklist [1]

SECTION	ITEM	PRISMA-ScR CHECKLIST ITEM	REPORTED ON PAGE #
**TITLE**			
Title	1	Identify the report as a scoping review.	Title page
**ABSTRACT**			
Structured summary	2	Provide a structured summary that includes (as applicable): background, objectives, eligibility criteria, sources of evidence, charting methods, results, and conclusions that relate to the review questions and objectives.	Title page
**INTRODUCTION**			
Rationale	3	Describe the rationale for the review in the context of what is already known. Explain why the review questions/objectives lend themselves to a scoping review approach.	Background
Objectives	4	Provide an explicit statement of the questions and objectives being addressed with reference to their key elements (e.g., population or participants, concepts, and context) or other relevant key elements used to conceptualize the review questions and/or objectives.	Background
**METHODS**			
Protocol and registration	5	Indicate whether a review protocol exists; state if and where it can be accessed (e.g., a Web address); and if available, provide registration information, including the registration number.	Methods
Eligibility criteria	6	Specify characteristics of the sources of evidence used as eligibility criteria (e.g., years considered, language, and publication status), and provide a rationale.	Methods – eligibility + Table 1
Information sources*	7	Describe all information sources in the search (e.g., databases with dates of coverage and contact with authors to identify additional sources), as well as the date the most recent search was executed.	Methods – search strategy
Search	8	Present the full electronic search strategy for at least 1 database, including any limits used, such that it could be repeated.	Methods – search strategy
Selection of sources of evidence†	9	State the process for selecting sources of evidence (i.e., screening and eligibility) included in the scoping review.	Methods – article selection
Data charting process‡	10	Describe the methods of charting data from the included sources of evidence (e.g., calibrated forms or forms that have been tested by the team before their use, and whether data charting was done independently or in duplicate) and any processes for obtaining and confirming data from investigators.	Methods – data extraction and synthesis

SECTION	ITEM	PRISMA-ScR CHECKLIST ITEM	REPORTED ON PAGE #
Data items	11	List and define all variables for which data were sought and any assumptions and simplifications made.	Methods – data extraction + Supp File 3
Critical appraisal of individual sources of evidence§	12	If done, provide a rationale for conducting a critical appraisal of included sources of evidence; describe the methods used and how this information was used in any data synthesis (if appropriate).	N/A
Synthesis of results	13	Describe the methods of handling and summarizing the data that were charted.	Methods – Data synthesis
**RESULTS**			
Selection of sources of evidence	14	Give numbers of sources of evidence screened, assessed for eligibility, and included in the review, with reasons for exclusions at each stage, ideally using a flow diagram.	Results – Included sources + Figure 1
Characteristics of sources of evidence	15	For each source of evidence, present characteristics for which data were charted and provide the citations.	Results – Included sources + Table 2 + Supp File 3
Critical appraisal within sources of evidence	16	If done, present data on critical appraisal of included sources of evidence (see item 12).	N/A
Results of individual sources of evidence	17	For each included source of evidence, present the relevant data that were charted that relate to the review questions and objectives.	Results – Attributes and impacts of CCCs + Primary research + changes in the literature over time + Table 3
Synthesis of results	18	Summarize and/or present the charting results as they relate to the review questions and objectives.	Results – Synthesis of attributes and effects of CCCs + Figure 3
**DISCUSSION**			
Summary of evidence	19	Summarize the main results (including an overview of concepts, themes, and types of evidence available), link to the review questions and objectives, and consider the relevance to key groups.	Discussion
Limitations	20	Discuss the limitations of the scoping review process.	Discussion
Conclusions	21	Provide a general interpretation of the results with respect to the review questions and objectives, as well as potential implications and/or next steps.	Conclusion
**FUNDING**			
Funding	22	Describe sources of funding for the included sources of evidence, as well as sources of funding for the scoping review. Describe the role of the funders of the scoping review.	Title page

Supplementary File S2. Database search strategies

Initial search: 5 Jan 2023 for all main databases (not including gray literature)

Note: While ESMO DCs and MASCC DCs were initially considered to be centers of similar concepts to CCCs, and search terms pertaining to these were included in the initial search strategy, decision was later made to exclude these from the review due to the differences revealed by the literature, where ESMO DCs are specifically focusing on palliative care (Cherny 2010) and MASCC DCs on supportive care (Ida 2021). Furthermore, it was acknowledged that although the initial plan was for ESMO DCs to follow an accreditation program based on the NCI CCC model, the concept of accrediting center of excellence was later modified as the latter criteria was deemed to be too restrictive, and many NCI CCCs have in fact applied to be recognized as ESMO DCs (Cherny 2010), highlighting the differences between CCCs and ESMO DCs that warranted separate certification programs for the two.

PubMed

Search	Query
#1	"Comprehensive cancer cent*"[tiab] OR "OECI CCC*"[tiab] OR "NCI CCC*"[tiab] OR "ESMO designated cent*"[tiab] OR "European Society of Medical Oncology designated cent*"[tiab] OR "Center of Integrated Oncology and Palliative Care"[tiab] OR "Centers of Integrated Oncology and Palliative Care"[tiab] OR "ESMO DC*"[tiab] OR "MASCC designated cent*"[tiab] OR "Multinational Association of Supportive Care in Cancer designated cent*"[tiab] OR "Center of Excellence in Supportive Care in Cancer"[tiab] OR "Centers of Excellence in Supportive Care in Cancer"[tiab] OR "MASCC DC*"[tiab]
#2	Limit publication date to 2002 onwards
#3	Limit to English Language

2381 results

Cochrane Library

Search	Query
#1	("Comprehensive cancer center" OR "Comprehensive cancer centers" OR "Comprehensive cancer center" OR "Comprehensive cancer centers" OR "OECI CCC" OR "OECI CCCs" OR "NCI CCC" OR "NCI CCCs" OR "ESMO designated center" OR "ESMO designated centers" OR "ESMO designated center" OR "ESMO designated centers" OR "European Society of Medical Oncology designated center" OR "European Society of Medical Oncology designated centers" OR "European Society of Medical Oncology designated center" OR "European Society of Medical Oncology designated centers" OR "Center of Integrated Oncology and Palliative Care" OR "Centers of Integrated Oncology and Palliative Care" OR "ESMO DC" OR "ESMO DCs" OR "MASCC designated center" OR "MASCC designated centers" OR "MASCC designated center" OR "MASCC designated centers" OR "Multinational Association of Supportive Care in Cancer designated center" OR "Multinational Association of Supportive Care in Cancer designated centers" OR "Multinational Association of Supportive Care in Cancer designated center" OR "Multinational Association of Supportive Care in Cancer designated centers" OR "Center of Excellence in Supportive Care in Cancer" OR "Centers of Excellence in Supportive Care in Cancer" OR "MASCC DC" OR "MASCC DCs"):ti,ab,kw.
#2	Limit publication date to 2002 onwards

276 results (all trials; 0 result for reviews)

CINAHL via EBSCO

Search	Query
#1	TI ("Comprehensive cancer cent*" OR "OECI CCC*" OR "NCI CCC*" OR "ESMO designated cent*" OR "European Society of Medical Oncology designated cent*" OR "Center of Integrated Oncology and Palliative Care" OR "Centers of Integrated Oncology and Palliative Care" OR "ESMO DC*" OR "MASCC designated cent*" OR "Multinational Association of Supportive Care in Cancer designated cent*" OR "Center of Excellence in Supportive Care in Cancer" OR "Centers of Excellence in Supportive Care in Cancer" OR "MASCC DC*") OR AB ("Comprehensive cancer cent*" OR "OECI CCC*" OR "NCI CCC*" OR "ESMO designated cent*" OR "European Society of Medical Oncology designated cent*" OR "Center of Integrated Oncology and Palliative Care" OR "Centers of Integrated Oncology and Palliative Care" OR "ESMO DC*" OR "MASCC designated cent*" OR "Multinational Association of Supportive Care in Cancer designated cent*" OR "Center of Excellence in Supportive Care in Cancer" OR "Centers of Excellence in Supportive Care in Cancer" OR "MASCC DC*")
#2	Limit publication date to 2002 onwards
#3	Limit to English Language

1451 results

EPISTEMONIKOS

Search	Query
#1	Title:("Comprehensive cancer center" OR "Comprehensive cancer centers" OR "Comprehensive cancer center" OR "Comprehensive cancer centers" OR "OECI CCC" OR "OECI CCCs" OR "NCI CCC" OR "NCI CCCs" OR "ESMO designated center" OR "ESMO designated centers" OR "ESMO designated center" OR "ESMO designated centers" OR "European Society of Medical Oncology designated center" OR "European Society of Medical Oncology designated centers" OR "European Society of Medical Oncology designated center" OR "European Society of Medical Oncology designated centers" OR "Center of Integrated Oncology and Palliative Care" OR "Centers of Integrated Oncology and Palliative Care" OR "ESMO DC" OR "ESMO DCs" OR "MASCC designated center" OR "MASCC designated centers" OR "MASCC designated center" OR "MASCC designated centers" OR "Multinational Association of Supportive Care in Cancer designated center" OR "Multinational Association of Supportive Care in Cancer designated centers" OR "Multinational Association of Supportive Care in Cancer designated center" OR "Multinational Association of Supportive Care in Cancer designated centers" OR "Center of Excellence in Supportive Care in Cancer" OR "Centers of Excellence in Supportive Care in Cancer" OR "MASCC DC" OR "MASCC DCs") OR Abstract:("Comprehensive cancer center" OR "Comprehensive cancer centers" OR "Comprehensive cancer center" OR "Comprehensive cancer centers" OR "OECI CCC" OR "OECI CCCs" OR "NCI CCC" OR "NCI CCCs" OR "ESMO designated center" OR "ESMO designated centers" OR "ESMO designated center" OR "ESMO designated centers" OR "European Society of Medical Oncology designated center" OR "European Society of Medical Oncology designated centers" OR "European Society of Medical Oncology designated center" OR "European Society of Medical Oncology designated centers" OR "Center of Integrated Oncology and Palliative Care" OR "Centers of Integrated Oncology and Palliative Care" OR "ESMO DC" OR "ESMO DCs" OR "MASCC designated center" OR

Search	Query
	“MASCC designated centers” OR “MASCC designated center” OR “MASCC designated centers” OR “Multinational Association of Supportive Care in Cancer designated center” OR “Multinational Association of Supportive Care in Cancer designated centers” OR “Multinational Association of Supportive Care in Cancer designated center” OR “Multinational Association of Supportive Care in Cancer designated centers” OR “Center of Excellence in Supportive Care in Cancer” OR “Centers of Excellence in Supportive Care in Cancer” OR “MASCC DC” OR “MASCC DCs”)
#2	Limit publication date to 2002 to 2023

587 results

PROSPERO

Search	Query
#1	Comprehensive cancer center OR Comprehensive cancer centers OR Comprehensive cancer center OR Comprehensive cancer centers

138 results

Updated search: 6 Oct 2023 for all main databases (not including gray literature)

PubMed

Search	Query
#1	“Comprehensive cancer cent*”[tiab] OR “OECI CCC*”[tiab] OR “NCI CCC*”[tiab]
#2	Limit publication date to 2023 onwards
#3	Limit to English Language

244 results

Cochrane Library

Search	Query
#1	(“Comprehensive cancer center” OR “Comprehensive cancer centers” OR “Comprehensive cancer center” OR “Comprehensive cancer centers” OR “OECI CCC” OR “OECI CCCs” OR “NCI CCC” OR “NCI CCCs”):ti,ab,kw.
#2	Limit publication date to 2023 onwards

12 results (all trials; 0 results for reviews)

CINAHL via EBSCO

Search	Query
#1	TI (“Comprehensive cancer cent*” OR “OECI CCC*” OR “NCI CCC*”) OR AB (“Comprehensive cancer cent*” OR “OECI CCC*” OR “NCI CCC*”)
#2	Limit publication date to 2023 onwards
#3	Limit to English Language

89 results

EPISTEMONIKOS

Search	Query
#1	Title:(“Comprehensive cancer center” OR “Comprehensive cancer centers” OR “Comprehensive cancer center” OR “Comprehensive cancer centers” OR “OECI CCC” OR “OECI CCCs” OR “NCI CCC” OR “NCI CCCs”)

Search	Query
	OR Abstract:(“Comprehensive cancer center” OR “Comprehensive cancer centers” OR “Comprehensive cancer center” OR “Comprehensive cancer centers” OR “OECI CCC” OR “OECI CCCs” OR “NCI CCC” OR “NCI CCCs”)
#2	Limit publication date to 2023

40 results

PROSPERO

Search	Query
#1	Comprehensive cancer center OR Comprehensive cancer centers OR Comprehensive cancer center OR Comprehensive cancer centers
#2	Limit date added to PROSPERO to 01/01/2023 to 6/10/2023

39 results

Updated search: 7 May 2024 for all main databases (not including gray literature)

PubMed

Search	Query
#1	“Comprehensive cancer cent*”[tiab] OR “OECI CCC*”[tiab] OR “NCI CCC*”[tiab]
#2	Limit publication date to 6 Oct 2023 onwards
#3	Limit to English Language

190 results

Cochrane Library

Search	Query
#1	(“Comprehensive cancer center” OR “Comprehensive cancer centers” OR “Comprehensive cancer center” OR “Comprehensive cancer centers” OR “OECI CCC” OR “OECI CCCs” OR “NCI CCC” OR “NCI CCCs”):ti,ab,kw.
#2	Limit publication date to 6 Oct onwards

26 results (all trials; 0 results for reviews)

CINAHL via EBSCO

Search	Query
#1	TI (“Comprehensive cancer cent*” OR “OECI CCC*” OR “NCI CCC*”) OR AB (“Comprehensive cancer cent*” OR “OECI CCC*” OR “NCI CCC*”)
#2	Limit publication date to Oct 2023 onwards
#3	Limit to English Language

63 results

EPISTEMONIKOS

Search	Query
#1	Title:(“Comprehensive cancer center” OR “Comprehensive cancer centers” OR “Comprehensive cancer center” OR “Comprehensive cancer centers” OR “OECI CCC” OR “OECI CCCs” OR “NCI CCC” OR “NCI CCCs”) OR Abstract:(“Comprehensive cancer center” OR “Comprehensive cancer centers” OR “Comprehensive cancer center” OR “Comprehensive cancer centers” OR “OECI CCC” OR “OECI CCCs” OR “NCI CCC” OR “NCI CCCs”)
#2	Limit publication date to 6 Oct 2023 to 13 May 2024

135 results

Supplementary File S3. Condensed data extraction table

Author, year, and country	Study design / description of source	Aim of study / source	Setting, and type of CCC focus	Population / cancer type focused on	Attributes of CCCs	Impacts
**Peer-reviewed publications**						
Ford 2004, USA [2]	Opinion	To discuss the role of CCCs in treating the whole person.	USA setting, accreditation body not discussed	Nonspecific, cancer in general	All cancer care located under one roof, 'one stop shopping'.<br><br>Provision of holistic care that is focused on the 'mind, body and spirit'.<br><br>Bringing together diagnostic, treatment and support services and the latest technology in a very nonclinical space.	**Provider level**<br>Comprehensive approaches lead to better and more coordinated care. Patients benefit from seeing their doctors talking to one another and working together and by being able to do everything in one visit.<br>CCCs are enjoyable places to work for staff.<br>Having all facilities in one place makes it easier for the patients.<br><br>**Organisational level**<br>The 4-story center combines all services including a center for integrative health and healing so patients can visit between appointments to help with wellbeing.
Ringborg 2008, Europe [3]	Commentary	To describe the rationale and aim of the OECI in developing CCCs, and collaboration between CCCs.	European setting, OECI designated CCCs	Nonspecific, cancer in general	The OECI is a non-profit organisation that has cancer centers/institutes as members, and has European Economic Interest Grouping, with a legal status in the European Union.<br>The mission of the OECI is to stimulate the development of CCCs in Europe and facilitate collaboration between the centers.<br>CCCs are organisations that integrate care and prevention, research, and education.	**Organisational level**<br>Goal of decreasing cancer-related morbidity and mortality and increasing survival and quality of life.

Author, year, and country	Study design / description of source	Aim of study / source	Setting, and type of CCC focus	Population / cancer type focused on	Attributes of CCCs	Impacts
					CCCs provide high-quality, patient-focused care throughout the clinical pathway including diagnostic activities; surgical specialities; radiation therapy and medical oncology; psychosocial oncology, rehabilitation, supportive care, and palliative oncology.<br>CCCs may have sufficiently developed structures for research and education.<br><br>Research in CCCs should include expertise in epidemiology and biostatistics and be translational, spanning preclinical and clinical, through to implementation and evaluation in clinical care.<br>Education in CCCs should be focused on all function in the center including basic education of nursing and medical students, specialist training, continuous medical education, research education, disease prevention education.<br><br>CCCs should provide decision makers with information about issues and strategies to improve cancer care.	

Author, year, and country	Study design / description of source	Aim of study / source	Setting, and type of CCC focus	Population / cancer type focused on	Attributes of CCCs	Impacts
					Ideally CCCs should not be fragmented within a university hospital – but be visible and identifiable as a CCC.<br>CCCs that reside in university hospitals have the benefit of geographical closeness with other disciplines of interest for cancer patients suffering with other diseases.	
Soo 2008, Singapore [4]	Case study	To describe the experience of developing a CCC in Singapore.	Singapore setting, no formal accreditation body discussed	Single CCC in Singapore (National Cancer Center Singapore). Focus on cancer in general.	The National Cancer Center has 3 important components: clinical service, research, and public education.<br>Clinical departments include medical oncology, radiation oncology, surgical oncology, palliative medicine, and oncological imaging.<br>Research divisions include clinical trials and epidemiological sciences, medical sciences, and cellular and molecular research.<br>The public education provides cancer education and an information service.<br>The National Cancer Center was built in the Singapore General Hospital complex. Amalgamation of the center had challenges but was	**Provider level**<br>Clinical and scientific staff train medical students in the 2 medical schools, and the center hosts local and overseas fellows, pre- and post-doctoral students, and other graduate students in biomedical sciences.

Author, year, and country	Study design / description of source	Aim of study / source	Setting, and type of CCC focus	Population / cancer type focused on	Attributes of CCCs	Impacts
					easily facilitated by all entities being in 1 geographical location.	
Pelagio 2011, Italy [5]	Review	To summarise the characteristics of a CCC biobank and multilevel procedures to maintain the biobank.	International setting - CCCs in general, accreditation body not discussed	Nonspecific, cancer in general	CCCs play a leading role in enabling the objectives of national health plans via research and education and training for healthcare professionals.<br><br>CCCs have administrative and operational autonomy and pursue both high-quality clinical care and translatable research.<br><br>CCCs participate in development of guidelines and provide technical and organisational support to national patient services.<br><br>CCCs have privileged access to biospecimens and longitudinal clinical data and are the ideal setting for biobanks due to the co-location of surgical and clinical services focused on cancer treatment.<br><br>Biobanks within CCCs are research-focused integrated service infrastructures that provide biospecimens and data, to understand long-term benefits and	**Organisational level**<br>As a result of this organizational model, CCC biobanks have a continuous stream of collection of samples and related information, unlike in individual research projects in which samples and annotation are collected only from selected candidates with specific requirements.

Author, year, and country	Study design / description of source	Aim of study / source	Setting, and type of CCC focus	Population / cancer type focused on	Attributes of CCCs	Impacts
					side effects of drugs and other treatments.	
Ali 2012, India and England [6]	Opinion	To describe the experience of a cancer research network in India, partnering with University of Oxford, England (INDOX)	Indian setting, CCCs in India (no formal accreditation system described)	Top 9 CCCs in India and University of Oxford, cancer in general	Provide practical and theoretical training in conducting Phase 1 trials.<br>Provided a comprehensive training program for all staff involved in clinical trials covering Good Clinical Practice.<br>Uniform quality standards with appropriate Standard of Practices implemented across all sites.<br>Dedicated clinical trials units and trained clinical research staff and are equipped to conduct trials to international standards.	**Provider levels**<br>More than 100 Primary Investigators, coordinators and research nurses etc. attended training courses in Oxford and India.<br><br>**Provider level**<br>High quality trial practice and adherence to Good Clinical Practice and Food and Drug Administration audits.<br>Brought many investigators together for the first time to jointly design and conduct clinical studies, developing relationships.<br>Appointed and trained dedicated site coordinators/research support officers.<br><br>**System/societal level**<br>Worked closely with Drugs Controller General of India to improve research in the country.
Clayman 2013, USA [7]	Qualitative – semi-structured interviews	To determine what fertility preservation resources are available in CCCs and how well those are integrated into patient care.	USA settings, NCI-CCCs	NCI-CCCs (n=30/39, 77% response rate), cancer in general	Fertility services on-site or referral programs.<br>Experimental services, such as ovarian tissue cryopreservation.<br>Time dedicated to fertility preservation or institutional policies about consistent provision of fertility information.<br>Resource-laden compared with community clinics.	

Author, year, and country	Study design / description of source	Aim of study / source	Setting, and type of CCC focus	Population / cancer type focused on	Attributes of CCCs	Impacts
Deriu 2013, Italy [8]	Position paper - review and opinion	To: 1) describe the most relevant elements of the accreditation models most used in Italy: and 2) define the recommendations for external quality control programs aimed at accreditation of excellence of Italian CCCs.	Italian setting, OECI designated CCCs	Network of 11 Italian CCCs, cancer in general	OECI CCCs are: highly innovative; have multidisciplinary characteristics, work to improve professional and organisational quality; continuously work to improve care for cancer patients; and perform activities in the fields of prevention, screening, research, education and popularisation of knowledge and innovation.	**Organisational level**<br>Undergoing an external quality verification has strong educational value and helps the improvement an of safety and quality procedures.<br>Institutions are recognised as research institutes based on quality certification according to internally recognised procedures.<br><br>**System/societal level**<br>Through networks, CCCs may collaborate within larger international programs.
Van Harten 2013, Europe and USA [9]	Opinion	To describe the role of CCCs in survivorship care.	Europe and USA setting, OECI designated CCCs and Association of American Cancer Institutes cancer research centers.	Nonspecific, cancer in general	CCCs play a key role in the field of survivorship care due to their strength in translational research, and provision of the full spectrum of treatments for patients throughout their clinical course.<br>CCCs can play a leading role in research and treatment development based on their multidisciplinary expertise in curing and providing supportive care for large numbers of patients with various conditions.<br>Some CCCs have explored models of survivorship clinics, to address long-lasting or late-onset effects of cancer treatment.	

Author, year, and country	Study design / description of source	Aim of study / source	Setting, and type of CCC focus	Population / cancer type focused on	Attributes of CCCs	Impacts
Eggermont 2014, Europe [10]	Opinion	To provide an overview of the Cancer Core Europe Alliance initiative, a research network of leading CCCs in Europe.	European setting, accreditation body not discussed	6 leading European CCCs, cancer in general	CCCs work together to enable cancer care-cancer research continuum by forming a consortium of six leading excellent comprehensive cancer centers.	**Provider level**<br>A network of CCCs provides unique environment to train next generation of staff to drive innovative translational and clinical oncology.<br><br>**System/societal level**<br>Networks of CCCs can meet the substantial demands of joint translational and clinical research programs, and provide the full spectrum of research (basic science to translational research).
Saghatchian 2014, Europe [11]	Observational – analysis of mixed methods accreditation data	To describe the landscape of the first 10 participating cancer centers in the OECI accreditation and designation program and describe their compliance with the standards of the OECI program.	European setting, OECI CCC designation	Cancer centers applying for OECI CCC designation, cancer in general	All 10 cancer centers applied for CCC designation; 5 were designated as CCCs, and 4 as clinical CCs (1 center was awaiting designation at the time of publication, pending major changes).<br>Criteria related to research shortcomings included lack of publications in journals with high impact factor lack of clinical trials, lack of integration of research into care or between laboratories. Care shortcomings mainly concerned lack of harmonization between patients and quality policy and guidelines. Lack of an identifiable dedicated integrated structure for cancer management was also a key issue in obtaining CCC designation,	

Author, year, and country	Study design / description of source	Aim of study / source	Setting, and type of CCC focus	Population / cancer type focused on	Attributes of CCCs	Impacts
					particularly in cancer centers in large university hospitals.<br>For 5 centers that failed to receive CCC designation 3 had research shortcomings, 1 had research and care issues, and 1 was related to care alone.	
Ancarani 2015, Italy [12]	Opinion	To describe the experiences of an institute which took part in the OECI accreditation program for CCCs, and how the process led to other opportunities, such as setting the foundations for the CCC Network	Italian setting, OECI designated CCCs	Non-specific, cancer in general	A network model comprising both major and complementary oncologic disciplines, would be under the direct responsibility of IRST.<br>Shared management with a board composed of directors from both entities.	**Provider level**<br>More complete approach to the care of the cancer patient through the integration of different disciplines from diagnosis to treatment and from follow-up to the management of critical phases.<br>Reduction in patient migration due to Innovative treatments available through the network with proximity to care.<br><br>**Organisation level**<br>Increased value of scientific production.<br>Higher accessibility to research funding.<br>Availability of hospital logistics platforms for high-complexity treatments.<br>Higher number of scientific collaborations.<br>Mutual exchange of information on clinical startup activities for high-complexity care in hospitals and scientific information.
Canitano 2015, Italy [13]	Commentary	To describe the improvement plan relating to 3 (out of 26 actions) actions following	Italian setting, OECI designated CCC	Single OECI designated CCC, caner in general	Establishment of clinical trial center to support and facilitate non-profit and profit trials.<br>Computerised ambulatory medical record - Integrated technology platform for outpatient care.	**Provider level**<br>Provides updated information for patients and researchers.<br>Coordinate research nurses.<br>Assure quality control of clinical trials.

Author, year, and country	Study design / description of source	Aim of study / source	Setting, and type of CCC focus	Population / cancer type focused on	Attributes of CCCs	Impacts
		OECI accreditation.			Centrality of the patient and humanisation of the clinical pathway.<br>Reviews of current patient services, organisation of patient empowerment network, promote application of narrative-base medicine, promote training courses that support patient centered care.	**Organisation level**<br>A more cohesive environment (e.g., the merging of various clinical areas into a single department and of experimental research and high technology).<br>Integrated healthcare information system within the institute allows sharing of medical examinations and data across all systems; standardization; better patient care and safety; reduction in waiting time for delivering medical records and waiting for outpatient services; and better management of cancer registry and research.
Da Pieve 2015, Italy [14]	Opinion	To describe an Italian CCCs experience of accreditation with OECI	Single OECI designated CCC	Single OECI designated CCC, caner in general	Multidisciplinary approach to care.<br>Integrated internal audits.<br>Organisational support for accreditation process.	**Provider level**<br>Patient involvement at every stage.<br><br>**Organisation level**<br>Comparability of data.
De Paoli 2015, Italy [15]	Commentary	To describe the rationale, development, and role of 'Alliance Against Cancer'.	Italian setting, OECI designated CCCs	6 CCCs in Italy, cancer in general	Consortium of 6 CCCs in Italy.<br>Engage in international partnerships.	**Provider level**<br>Generation of synergies with other specialists involved in health care to increase coverage of a greater extent of rarer types of cancers.<br><br>**Organisation level**<br>Development of specific advanced projects in clinical and translational research.<br>Bidirectional information exchange between patients and institutes with a continuous stimulus to look after patient's needs, when cancer patient organisations are included in the networks.<br><br>**System/societal level**

Author, year, and country	Study design / description of source	Aim of study / source	Setting, and type of CCC focus	Population / cancer type focused on	Attributes of CCCs	Impacts
						Promote a network among Italian CCCs to develop specific, advanced projects in clinical and translational research.<br>Engagement in international partnerships to facilitate the development of innovative clinical trials and increase the numbers of eligible patients.<br>Achievement of high standards of care and harmonisation of principles across country.<br>Increasing of the number of clinical trials and offering of innovative therapies to patients all around the country.
Hammer 2015, USA [16]	Observational – survey	To provide an updated assessment regarding supportive care services and subjectiveness effectiveness of such services (comparing changes that have occurred over a 17-year period).	USA setting, NCI-CCCs and CCs	NCI CCCs and NCI-CCs (n=31/41, 76% response rate). Focus on cancer in general.	Integration of supportive care services, availability of complementary services, and the number of pain and palliative care services have increased over the past 17 years.<br>There has been an increase in the number of centers that offer pain management services. Pain management was offered by all centers, followed by nutritional counselling (88%), a palliative care clinic (88%), ostomy care (76%), and rehabilitation (72%).<br>Other Attributes included: Navigation (96%) and advocacy (92%). The following programs relaxation/meditation, guided imagery, art therapy,	**Provider level**<br>Increased use of Distress Screening used by 64%.<br><br>**Organisational level**<br>A total of 54% of institutions reported collecting evaluation data on their programs.

Author, year, and country	Study design / description of source	Aim of study / source	Setting, and type of CCC focus	Population / cancer type focused on	Attributes of CCCs	Impacts
					family/caregiver programs, and bereavement (84%).<br>Genetic counselling (81%).	
Platek 2015, USA [17]	Observational – survey	To determine the prevalence and types of outpatient clinical nutrition services available at NCI CCCs.	USA setting, NCI-CCCs	Telephone survey at NCI CCCs n=32/40 (80% response rate) with registered dieticians. Focus on cancer in general.	Most (94%) CCCs had referral or consultative based services for outpatients with a nutrition profession such as a registered dietician.<br>Three quarters (73%) of the CCCs monitored outpatients regularly, but only half (48%) followed a clinical nutrition protocol.<br>Specific nutritional services were provided at 56% and 46% of CCCs for head and neck and gastrointestinal cancers, respectively.	**Provider level**<br>For the centers that provide clinical nutrition services via a referral or consult system (n=30), 23 said that they followed referred patients regularly.<br>91% stated that the protocol was part of standard of care.<br>15 centers provided head and neck patients who were referred with regular follow-up. Of these 15, seven followed a specific evidence-based protocol, and six out of the seven incorporated these protocols into standard of care.
Ricciardi 2015, Italy [18]	Commentary	To describe the results of a special national program called Istituto Speriore di Sanita, that aimed to support a network of CCCs in Italy (Alliance Against Cancer).	Italian setting – network of CCCs, no accreditation body discussed.	Network of 11 CCCs – Alliance Against Cancer. Focus on cancer in general.	The establishment of the network of CCCs (Alliance Against Cancer), to avoid fragmentation and foster common strategies for enhancing the quality of oncology research and care at the national level, with a view to international collaboration.	**System/societal level**<br>The program has led to several joint initiatives at the national and European level including increased cooperation among European members states for funding and high-quality translational projects.

Author, year, and country	Study design / description of source	Aim of study / source	Setting, and type of CCC focus	Population / cancer type focused on	Attributes of CCCs	Impacts
Berendt 2016, Germany [19]	Qualitative – Delphi study	To develop consensus-based best practice recommendations for the integration of palliative care in German CCCs	German setting, CCCs certified by the German Cancer Society and funded by the German Cancer Aid	Multi-professional experts (N=52) from 15 German CCCs, cancer in general	Leading role in clinical care, education and research in Germany.<br>Serve as models that may influence cancer care in general.<br>Uphold the highest possible performance standards caring for the most complex cancer patients.<br>Have an inpatient palliative care consultation service and an outpatient palliative care clinic.	
Hamlyn 2016, USA[20]	Mixed methods – mystery shopper assessment	To identify and quantify barriers faced by patients seeking to make a first consultation appointment with NCI-CCCs	USA setting, NCI-CCCs (N=40)	Mystery shoppers posing as people with, or children of people with newly diagnosed cancer	There was no statistically significant variation between appointment availability for people across various insurance types.<br>Callers who reported having Medicaid insurance had longer wait times until first appointment, and reported differences in experience in qualitative data (i.e., told that CCCs did not take patients with Medicaid insurance).	
Rajan 2016, Netherlands, Finland, UK [21]	Commentary and pilot	To describe an Excellence Designation System for translation of research from the bench to the bedside, and back.	International setting (Europe and United Kingdom), OECI designated CCCs	Nonspecific focus, and 3 CCCs as pilot sites. Focus on cancer in general.	Non-Hierarchical committed team, excellent mentoring, cost-effective home-grown innovations, biobanking facilities.<br>Criteria for excellence in translational research was developed that covers articulation of a vision, experience and commitment, tangible evidence of	**Organisational level**<br>High patient accrual in trials.<br><br>**System/societal level**<br>Detailed demonstration of ongoing availability and clearly articulated intention to leverage funding and /or resources obtained due to 'excellent' designation.

Author, year, and country	Study design / description of source	Aim of study / source	Setting, and type of CCC focus	Population / cancer type focused on	Attributes of CCCs	Impacts
					outcome, sharing of resources, peer review, training programs, biobank/s, bench-to-bedside research, innovation, and consumer engagement.	
Yun 2017, USA [22]	Observational – website review	To determine the growth of integrative medicine in leading academic cancer centers in the USA as reflected by their public-facing websites.	USA setting, NCI-CCCs	Websites of NCI CCCs (N=45). Cancer in general	Between 2009 and 2016, NCI CCCs increasingly present integrative medicine content on their websites, and the majority of them provide these services to patients in the same health systems.<br>On the 45 NCI CCCs' websites, the most common integrative medicine therapies mentioned were exercise (97.8%), acupuncture and meditation (88.9% each), yoga (86.7%), massage (84.4%), and music therapy (82.2%). Most websites provided information on nutrition (95.6%), dietary supplements (93.3%), and herbs (88.9%).<br>The most common therapies offered in CCCs were acupuncture/massage (73.3% each), meditation/yoga (68.9% each), and consultations about nutrition (91.1%), dietary supplements (84.4%), and herbs (66.7%).	**Provider level**<br>Meeting patients' information-seeking needs ensures that cancer patients use evidence-informed complementary therapies together with, rather than instead of, conventional.<br>treatments.

Author, year, and country	Study design / description of source	Aim of study / source	Setting, and type of CCC focus	Population / cancer type focused on	Attributes of CCCs	Impacts
Adami 2018, European countries [23]	Opinion	To present the viewpoints of the EACS regarding the cancer research continuum and highlights specific areas where initiatives should be taken	European setting, designated CCCs by OECI or the German Cancer Aid	Non-specific, cancer in general	Link research with the healthcare system.<br>Provide multidisciplinary academic expertise.<br>Integrate prevention strategies.<br>Interaction with CCs regarding the quality of care, innovation, and research collaboration.	**Provider level**<br>Rapidly incorporate innovations with rigorously demonstrated benefits for patients into clinical guidelines.<br>Integrate multidisciplinary cancer care with research and education.<br><br>**Organisational level**<br>Provide the environment for translational research in which basic cancer researchers interact closely with clinical colleagues in a mutually stimulating academic environment.<br>The ability to rapidly recruit an adequate number of patients with specific biomarker signatures are integral in translational research.<br><br>**System/societal level**<br>Close collaboration between CCCs provides the full range of expertise and resources, and access to an adequate number of patients (for innovative research and personalised/precision cancer medicine).
Rolland 2018, USA [24]	Observational – review and analysis of websites	To understand the types of posttreatment survivor-specific resources available on CCCs' websites.	USA setting – NCI designated	Websites of NCI CCCs (N=47), cancer in general	Although 75% (n=35) of CCCs had some information on their websites, limited survivor-specific services information was available for patients, caregivers or clinicians.<br><br>45% (n=15) CCCs websites had explicit information on surveillance; 36% (n=17%) discussed prevention activities; 43% had information	

Author, year, and country	Study design / description of source	Aim of study / source	Setting, and type of CCC focus	Population / cancer type focused on	Attributes of CCCs	Impacts
					regarding survivor-specific mental health; 40% had any reference to survivorship cancer plans; and 51% offered information about a general survivorship program.<br><br>NCI CCCs serve as a model to community oncologists and clinics in the delivery of best-practice survivorship care.<br>CCCs are expected to be leaders for community providers, and function as sources of information for survivors, caregiver and clinicians in navigating care after treatment.	
Brandts 2019, Germany [25]	Opinion	To provide a comprehensive view on how outreach can be achieved in CCCs to include all stakeholders to assure quality in cancer care and create innovation, as well as to integrate decentralised versus	International setting, accreditation body not discussed	Non-specific, cancer in general	New diagnostic methods not readily available to outreach partners.<br>Conduct basic and translational cancer research.<br>Standard operating procedures (SOPs) based on international guidelines to harmonize the diagnostic and therapeutic procedures.<br><br>Interdisciplinary education and training programs.<br>Supported weekly tumor boards by attending these physically or virtually.	**Provider level**<br>Involve all professional groups (including clinicians, scientists, nurses, administrative staff, students, and patients).<br><br>**Organisational level**<br>Integration of all departments and institutes responsible for patient care, education, and cancer research.<br><br>Stronger integration of translational cancer research approaches.<br><br>Increase in clinical trial activity.

Author, year, and country	Study design / description of source	Aim of study / source	Setting, and type of CCC focus	Population / cancer type focused on	Attributes of CCCs	Impacts
		centralised patient care, education, and cancer research.			Information seminars of high quality offered to patients, their families, and self-help and patient advocacy groups, regardless of where their patients are treated.	
Eggermont 2019, Europe [26]	Opinion	To discuss the Cancer Core Europe Alliance and Cancer Prevention Europe and how they are working together to address the burden of cancer.	Europe setting (Cancer Core Europe Alliance), accreditation body not discussed	7 leading European CCCs, cancer in general	Cancer Core Europe links centers with robust basic/preclinical research with those having an outstanding experience in early phase clinical trials to address the translational research continuum.<br>Cancer Core Europe is a shared research infrastructure with research collaborations and task forces.<br>Standardizing and developing medical imaging technologies and image analysis.<br>Clinical and translational research with a focus on innovative precision cancer medicine - manages many specific subgroups of tumors characterized by individual molecular characteristics, and thus offers new possibilities to stratify patients for treatment.	**Provider level**<br>Increased patient empowerment through a synergistic relationship with patients to provide information, complete awareness, and participation in research projects, treatment decisions, and follow-up of care and outcomes, and therefore contributing to patient empowerment.<br><br>**Organisational level**<br>Common clinical molecular profiling is shared by all institutes and linked to a data center, which enables computational biology to provide validated diagnostic information; in addition, standard operating procedures are currently under development for tissue and liquid biopsies.<br>Common clinical databases allow assessments of clinical effectiveness and outcomes on a longitudinal term based on health-economics studies.<br><br>**System/societal level**<br>Moving towards a single e-hospital with shared translational research activities and compiled databases.
Joos 2019,	Opinion	To discuss the German Cancer Consortium.	German setting, German	Science and research	Engaged in broad portfolios of translational and clinical cancer research, with partner site-specific	**Organisational level**

Author, year, and country	Study design / description of source	Aim of study / source	Setting, and type of CCC focus	Population / cancer type focused on	Attributes of CCCs	Impacts
Germany [27]			CCCs (accreditation body not discussed)	centers and CCCs at 7 sites across Germany. Focused on cancer in general.	research and clinical profiles providing a solid basis for complementarity.<br>DKTK was constituted as a foundation under public law.<br>Funded on a long-term, institutional basis and competitive joint funding program.	Central hub for collecting and exchanging clinical data and biomaterials which provides a bridge across disciplines and<br>Institutions.<br><br>**System/societal level**<br>Contributes to several highly relevant areas of modern cancer research, including identification of molecular biomarkers for tumor classification risk-adapted stratification and new treatment paradigms.
Kim 2019, USA [28]	Observational – analysis of academic output	To examine the influence of Surgical Society Oncology membership with NCI status on the academic output of surgical faculty at NCI accredited CCCs and CCs.	USA setting, NCI-CCCs	Surgeons (n=4,015) at top 50-ranked university based and top 5-ranked hospital-based NIH funded departments for surgery (n=29 NCI CCCs, n=12 NCI CCs, n=13 non-NCI centers). Focus on	NCI CCC designation and Surgical Society Oncology membership had synergistically effect on increased citations and citations.	**Provider level**<br>At CCC, 22.7% of surgical faculty had a history of or current NIH funding, compared with 15.8% at the NCI CC and 11.8% at the non-NCI centers.<br>CCC surgical faculties were better funded by NIH R01/P01/U01 grants (9.5%) compared with those from NCICC (7.9%) and non-NCI centers (6.8%). CCC (11%) and non-NCI (8.5%) faculty were more likely to have SSO membership than at NCICC (4.6%), p <0.05.<br>CCCs had a trend for more surgical faculty with PhDs or MD-PhDs (12%) versus those at NCICC (6.5%) and non-NCI-centers (9.9%).<br><br>**Organisation level**<br>CCCs were more likely to have surgical faculty in leadership positions (13.7%) compared with NCICC (7.9%) and non-NCI centers (10%), p <0.05.

Author, year, and country	Study design / description of source	Aim of study / source	Setting, and type of CCC focus	Population / cancer type focused on	Attributes of CCCs	Impacts
				cancer in general.		
Oberst 2019, Europe [29]	Review	To summarise the organisational structures and processes essential for producing quality outcomes for patients and effectiveness in translation in CCCs.	European setting, OECI and EACS designated CCCs	Nonspecific, cancer in general	Key hallmarks of CCCs include:<br>Excellence in diagnosis, treatment, and patient care, through multidisciplinary teams.<br>High-quality outpatient and inpatient facilities, leading to excellent patient experience.<br>Strong clinical trials infrastructure and active/open clinical trials (led by CCC staff), with high recruitment rates of new patients.<br>High-quality diagnostics, and capabilities in molecular pathology, imaging, and histopathology.<br>Excellent e-hospital and information systems, supporting collection of clinical data and linkage for research.<br>Comprehensive education programs for clinicians, scientists, and patients/carers.<br>Commitment to networking across catchment area to serve population.<br>Integration with national cancer prevention, screening, and early detection strategies.<br>Access to high-quality basic science regarding cancer.	**Provider level**<br>Evidence of innovation through patents, spin-off companies, and practice change.<br><br>**Organisational level**<br>Integration seen in CCCs can lead to impacts around bridging translational research gaps.<br>Integrated Practice Units in CCCs can lead to increased clinical effectiveness and reduced costs, by the team taking responsibility for the whole patient pathway and monitoring data points and patient outcomes.<br>Career advancement opportunities for staff.<br>Consistent academic outputs for wide range of disciplines in highly rated journals.<br><br>**System/societal level**<br>Translation of research from preclinical science to clinical implementation.

Author, year, and country	Study design / description of source	Aim of study / source	Setting, and type of CCC focus	Population / cancer type focused on	Attributes of CCCs	Impacts
Berns 2020, Europe [30]	Opinion	To provide an update and detailed view of infrastructure requirements to promote excellence in cancer research in Europe and priority areas to realise Europe's 'cancer mission'	Countries in Europe (European Union members states), accreditation body not discussed	Non-specific, cancer in general	Availability of sufficiently large numbers of diverse patient groups for clinical research and access to large numbers of healthy subjects.<br>Molecular pathology including multi-omics technologies and immunotyping.<br>State-of-the-art infrastructure for early clinical trials, next-generation clinical trials, practice changing clinical trials and implementation research.<br>Follow-up monitoring/treatment adaptation by repeated biopsies and functional/molecular imaging technologies.<br><br>CCCs part of integrated, networked, and geographically distributed organisations including CCCs, CCCs of 'excellence', cancer researchers, CCs, and technological platforms.	**Organisational level**<br>Ability to stratify patients and healthy subjects for distinct treatment arms through molecular pathology.<br><br>**System/societal level**<br>Access to a critical mass of patients, biological materials and technological resources, bridging research and health care.
Gahr 2020, Germany [31]	Observational – survey	To evaluate the implementation of best practice recommendations for the integration of palliative care in CCCs.	German setting, OECI designated CCCs	Director of CCCs (n=15/15, 100% response rate), cancer in general	The majority of the German CCCs already fulfilled essential organizational and structural requirements of the Palliative Medicine Working Group guidelines.<br>Variation existed around availability of various palliative care services.	**Provider level**<br>Integration of best practice recommendations means that palliative care is offered to all patients after diagnosis of incurable cancer.<br><br>**Organisational level**<br>Tandem partnerships establish good networks between CCCs.

Author, year, and country	Study design / description of source	Aim of study / source	Setting, and type of CCC focus	Population / cancer type focused on	Attributes of CCCs	Impacts
					All CCCs (n=15) had a palliative care unit. 13/15 units had palliative care specialists available 24 hours a day. 11/15 CCCs offered specialist palliative care within inpatient oncology departments. 9/15 CCCs had at team of at least 3 multidisciplinary clinicians (medical, nursing, allied health). 12/15 CCCs had facilities for specialist palliative care in oncology outpatients. 11/15 had outpatient palliative care clinics. All CCCs had specialist palliative home care available. 11/15 CCCs enquire about living wills and power of attorney on admission. 9/15 CCCs submit data to a National Hospice and Palliative Care Registry. 6/15 CCCS had a quality concept for managing patients at the end of life (i.e., pathways). 12/15 CCCs had palliative medicine integrated into research structures of CCC. 10/15 CCCs have a structural concept to support research and teaching in the field of palliative medicine. 5/15 CCCs had ESMO certification.	
Pasick 2020, USA [32]	Qualitative –	To explore the feasibility and benefit of second	CCCs and community	African American women		**Provider level**<br>In 'second option' consultations, CCC clinicians offered valuable recommendations including changing

Author, year, and country	Study design / description of source	Aim of study / source	Setting, and type of CCC focus	Population / cancer type focused on	Attributes of CCCs	Impacts
	ethnographic	opinions from breast oncologists within CCCs for African Americans treated at community hospitals.	hospitals, NCI-CCCs	with breast cancer (N=14). Focus on breast cancer.		or modifying treatment plans and/or improving management of side effects. All second opinion recommendations were followed by treating clinicians at non-CCC hospitals.<br>Second opinions from oncologists at CCCs is feasible and can improve treatment quality.
Desai 2021, USA [33]	Observational – systematic review of cancer center websites	To compare the availability of integrative medicine therapies in NCI-designated CCCs, and community hospitals	USA setting, NCI-CCCs and CCs	NCI-CCCs (n=51) and community hospitals (n=100), cancer in general	Availability of acupuncture, meditation, and music therapy was significantly lower at community hospitals compared with CCCs. Community hospitals offered fewer integrative medicine therapies as compared with Comprehensive Cancer Centers.<br>Availability of acupuncture (56% vs. 76.5%, p = 0.01), meditation (63% vs. 82.4%, p = 0.02), and music therapy (55% vs. 74.5%, p = 0.02) was significantly lower at community hospitals compared with Comprehensive Cancer Centers.<br>For massage (80% vs. 84.3%, p = 0.52), yoga (79% vs. 84.3%, p = 0.43), fitness (72.6% vs. 85%, p = 0.07), and Tai Chi (45% vs. 51%, p = 0.49), there was no significant difference between community hospitals and CCCs.	

Author, year, and country	Study design / description of source	Aim of study / source	Setting, and type of CCC focus	Population / cancer type focused on	Attributes of CCCs	Impacts
Doykos 2021a, USA [34]	Commentary	To report highlights of a meeting to discuss health equity agenda of NCI (as led by Community Outreach and Engagement program) and identify areas for improvement.	National meeting in USA, NCI-CCCs	Meeting attended by current, emerging, and affiliated NCI CCCs, affiliated programs, broader cancer community. Focus on cancer in general.	Community Outreach program in NCI CCCs include:<br>Adopting and adequately resourcing an explicit health equity approach; understanding and addressing structural barriers to equitable cancer outcomes; improving access to and quality of care by streamlining patients' pathways across clinical settings; advancing relevant policies that support cancer control; effective evaluation.	
Doykos 2021b, USA [35]	Commentary	To report key themes from a meeting about community outreach programs.	USA setting, NCI-CCCs	22 NCI-CCCs and the broader community, cancer in general. Focus on cancer in general.	Identifying and understanding disparities by analysing data disaggregated by race and ethnicity as well as other demographics.<br><br>Formal mechanisms to regularly gather community input, and importantly, to share back research findings with the community.<br>Conducting implementation science research to inform improvements in care and treatment.<br>Partnerships with community-based health care organizations.	**Provider level**<br>Streamline care pathways and provide more holistic care.<br>Increased clinical trial enrolment of minority populations.<br>Increased clinical quality outcomes, including decreased time to resolution of cancer diagnoses.<br><br>**Organisational level**<br>Strengthening of community outreach capacity by working through community health educators located at NCI Cancer Centers.<br>Provide education, initial cancer screenings, streamlined referral systems between primary care providers and oncology specialists, second opinion

Author, year, and country	Study design / description of source	Aim of study / source	Setting, and type of CCC focus	Population / cancer type focused on	Attributes of CCCs	Impacts
					CCC Staff embedded within a local cancer hospital and a community hospital.	and treatment plan confirmation services and navigation.<br>Lend expertise to further the efforts of existing coalitions and engaging in policy to reduce disparities in prevention.<br><br>**System/societal level**<br>Expanding screening programs and the supportive services needed to make them effective.
Kehrloesser 2021, Europe [36]	Observation – mixed methods secondary analysis of accreditation data	To identify the hallmarks common to all cancer centers and the distinctive features of CCCs using OECI accreditation data.	European setting, OECI designated CCCs	OECI accredited CCCs and CCs (N=40). Focus on cancer in general.	Compared to CCs:<br>CCCs managed double the number of patients and had larger oncology budgets.<br>CCCs had higher volume, quality, and integration of translational research.<br>CCCs had better compliance in leadership and management, and research, innovation, and development.<br>The largest absolute difference between CCCs and CCs was organisational structure and governance.	**Organisational level**<br>CCCs had a median total publication output of 370 per year, compared with 104 for CCs.<br>Significant difference in the number of clinical trials open to recruitment in CCCs compared with CCs.<br>Higher patient numbers being recruited to prospective interventional trials per index year compared with median.<br><br>**System/societal level**<br>Median budget for oncology care was more than twice at CCCs (median €150.1 M) compared with CCs (median €68.4 M.<br>CCCs were significantly stronger than CCs in research collaborations, organisation of clinical research, processes of intellectual property and innovation, and infrastructure for biobanking.<br>CCCs were also more consistent in having a robust scientific knowledge transfer program, being subject to regular external review, and in engaging an international Scientific Advisory Board.

Author, year, and country	Study design / description of source	Aim of study / source	Setting, and type of CCC focus	Population / cancer type focused on	Attributes of CCCs	Impacts
Mueller 2021, USA [37]	Observational – review of NCI administrative data	To summarise the characteristics of NCI-funded dissemination and implementation grants in CCCs and CCs to understand the nature, extent, and opportunity for this type of translational work.	USA setting – data from National Institutes of Health Research Portfolio Online Reporting Tool. NCI-CCCs and CCs.	CCCs (n=51), CCs (n=13), and active affiliates. Focus on cancer in general.	There is considerable room for development to support. the NCI's mission to support translation of research.<br>62% of CCCs (n=32/51) and 38% of CCs (n=5/13) held a dissemination or implementation grant.	**Provider level**<br>Half of the grants focused on specifical cancers, most commonly colorectal, breast and cervical. Grants that were not focused on specific cancer focused more generally on health behaviour, community outreach, or health information technology.<br><br>**System/societal level**<br>Almost two thirds of the grants focused on health equity. The most common health equity topics were: 1) social, economic, or structural determinants of health; 2) race or ethnicity; 3) social needs; 4) socioeconomic status or income; and 4) rurality.
Ringborg 2021, Europe [38]	Commentary – summarising research summit	To summarise the key priorities and specific action points required for successful implementation of the European Cancer Mission and Europe's Beating Cancer Plan, as discussed at the European Cancer Research Summit in Porto 2021.	European setting, OECI designated CCCs	Nonspecific, cancer in general	High-quality networks of CCCs should focus on reducing cancer incidence, increasing cure rates, survival times and quality of life, and addressing care inequalities in Europe.<br>CCC networks should include translational research, clinical trials and outcomes research.<br>CCCs should integrate prevention, clinical care, and research across the entire cancer continuum to enable the development of personalised/precision cancer medicine.	

Author, year, and country	Study design / description of source	Aim of study / source	Setting, and type of CCC focus	Population / cancer type focused on	Attributes of CCCs	Impacts
					CCCs are organisations where research, care and education align and are fully integrated.<br>CCCs ideally should have program structure, linking clinicians and researchers around scientific topics or tumor groups or scientific topics.<br><br>CCCs pioneer innovation, implement new therapeutic pathways and can address inequalities by developing networks around them geographically.<br><br>CCCS are pivotal to delivering the Cancer Mission and the Europe's Beating Cancer Plan.	
Kalra 2022, USA [39]	Observational – description of service design and delivery	To describe an oncologist-only question and answer website that gathers document expert insights from Tumor Boards at NCI CCCs to provide educational benefits to the oncology community.	USA setting, NCI-CCCs	16 NCI CCCs, cancer in general.	Tumor Boards as standard practice. Experts at the NCI CCCs share experiential knowledge and best practices into actionable information.	**Provider level**<br>Between December 2016 and July 2021, a total of 534 answers to 368 questions have been posted from these 23 programs from 16 NCI CCC sites.<br>Q&As were viewed 147,661 times by the oncologists at 3515 institutions.<br><br>**Organisational level**<br>Excellent opportunities for experts to share knowledge.

Author, year, and country	Study design / description of source	Aim of study / source	Setting, and type of CCC focus	Population / cancer type focused on	Attributes of CCCs	Impacts
Kirtane 2022, USA[40]	Retrospective cohort study	To examine the timing of patients' presentation at an NCI-CCC relative to their diagnosis and demographic characteristics.	USA setting, single NCI-CCC	Patients with breast, colon, lung, melanoma, and prostate cancer who presented to a single NCI-CCC between 2008-2020	African American patients had a longer time between diagnosis and presentation to the NCI-CCC compared to White patients (median 510 vs 368 days).<br>African American patients were also more likely to have received their initial cancer care outside of the NCI-CCC compared to White patients (odds ratio 1.45, 95% confidence interval 1.32–1.60).<br>Furthermore, Hispanics were more likely to present to the NCI-CCC at an advanced stage compared to non-Hispanic patients (Odds ratio 1.29, 95% 1.05– 1.55).	
Lawler 2022, Europe [41]	Commentary	To provide evidence on the current landscape of cancer research across Europe and explore gaps and inequalities to guide future research agendas.	European setting, OECI designated CCCs	Nonspecific, cancer in general	CCC infrastructure must be underpinned by quality standards and accreditations for care and research.<br>CCCs have larger research budgets and see higher throughput of prospective interventional clinical trials (compared to CCs or non-accredited centers).<br>CCCs have higher availability of resources to support research such as patient numbers, protected time for academic clinicians, and support from research nurses, coordinators, and other professionals.	**Organisational level**<br>Critical mass and integration in CCCs are important for high-quality clinical trials.<br><br>**System/societal level**<br>CCCs have a key role to play in European cancer research and care agendas.<br>The European Union's Cancer Mission Board has recommended the establishment of a network of CCCs across member states to improve quality of care and research.<br>Several European networks of CCCs have been formed to address specific research areas and translation into clinical practice (such as precision oncology).

Author, year, and country	Study design / description of source	Aim of study / source	Setting, and type of CCC focus	Population / cancer type focused on	Attributes of CCCs	Impacts
Majumdar 2022, USA[42]	Observational – mixed methods (semi-structured interviews and surveys)	To identify a possible model to explain how merging teams and professions into a unified CCC might influence healthcare team processes and experience, and patient experience.	Single NCI-CCC	Health care professionals employed at an NCI-CCC (n=20/42, 48% response rate) including medical, nursing, allied health, and administrative staff.<br><br>Patients receiving outpatient cancer treatment in the hospital (n=50/50, 100% response rate), n=26, 52% male	The following were reported as positive features of merged CCCs that contribute to optimal performance of teams:<br>balanced workloads; component, knowledgeable and supportive co-workers; approachable and friendly work environment; availability of resources for employees to learn and grow, including new equipment with latest technology; interprofessional collaboration; structured and supportive leadership, strong professional connections, and cross-training practices.	**Provider level**<br>Improved patient outcomes and quality of care, improved ability to disseminate knowledge and improved patient satisfaction.<br>'Happy' employees, satisfied employees.<br>Collaborative learning from/with each other.<br><br>**Organisational level**<br>All clinics under one roof served as a one-stop shop for patients and reduced travel time between clinics/units.<br>Increased engagement in adaptive behaviour, enactment of organisational citizenship behaviour.

Author, year, and country	Study design / description of source	Aim of study / source	Setting, and type of CCC focus	Population / cancer type focused on	Attributes of CCCs	Impacts
				participants).		
Alaniz 2023, USA[43]	Observational – online survey	To explore the impact of Community Outreach and Engagement component has on the overall Cancer Center Support Grant merit descriptors and score for NCI-CCCs and CCs.	NCI-CCCs and CCs across USA	NCI-CCCs and CCs N=48/62 (77% response rates)	NCI-CCCs perform research that is relevant to their catchment areas, guided by data assessing and monitoring the cancer burden in the catchment area.<br><br>Community Outreach and Engagement component merit descriptors are strongly correlated (Spearman's rank correlation coefficient r= 0.544, p=0.0003) with Cancer Center Support Grant scopes for CCCs (but not CCs).	CCCs that score better in Cancer Center Support Grant applications may receive more funding or be eligible for extended renewal cycles. This indicates that Community and Outreach Engagement initiatives are an important investment for NCI-CCCs.<br><br>CCCs engaging and partnering with local communities can address health disparities and inequities in cancer, facilitate cancer outreach activities (i.e., education and screening) and support research projects that link the CCCs priorities directly with community needs.
Berns 2024, Netherlands[44]	Commentary	Discussion paper around the need for academia and society to make anti-cancer treatments more affordable	European setting	Nonspecific, cancer in general	CCCs combine high-quality basic research with the capacity to clinically explore innovative concepts, (facilitating bench-to-bed and bed-to-bench research), may be best placed for bringing new promising laboratory findings to the clinical.<br><br>CCCs and their networks are instrumental in facilitating strategies on how to make translational cancer research more effective, and high-quality cancer care and prevention more accessible and affordable for all citizens.	

Author, year, and country	Study design / description of source	Aim of study / source	Setting, and type of CCC focus	Population / cancer type focused on	Attributes of CCCs	Impacts
					CCCs of Excellence (designated through European Academy of Cancer Science Excellence Designation System) can play an indispensable role in the research continnum.<br><br>Through collaboration with other centers and hospitals, CCCs of Excellnce can develop new therapies, and expedite treatments through the cancer research continuum by guaranteeing swift recruitment, overseeing clinical trials, and providing benefit/cost analysis.	
Fervers 2024, France[45]	Commentary	To describe key prevention services in CCC in France	French setting, single CCC and surrounding region	Nonspecific, cancer in general	CCCs can contributes to overcoming the complex and multifaceted challenges of cancer prevention through its comprehensive cancer prevention strategy, grounded in clinical prevention services, outreach activities, cross-disciplinary research, and collaboration with decision makers and professional and territorial health communities.<br><br>Research is embedded at all stages of the prevention continuum, from	Prevention can be integrated into the comprehensive mission of CCCs, and CCCs can may contribute to bridging the current fragmentation between cancer care and the different components of primary, secondary, and tertiary prevention.<br><br>Due to increasing cancer incidence, the shift toward integrated prevention-centered cancer care is needed to improve population health and respond to the shortage of hospital staff and overcrowding in cancer services, as well as offer opportunities to reduce carbon emissions from cancer care.

Author, year, and country	Study design / description of source	Aim of study / source	Setting, and type of CCC focus	Population / cancer type focused on	Attributes of CCCs	Impacts
					understanding cancer causes through to implementation of prevention interventions during and after cancer.<br><br>Health promotion activities in the community and dedicated outpatient primary cancer prevention services are provided for individuals at increased risk.	
Odedina 2024, USA[46]	Qualitative – impact and logic models	Describe the development of two guide models that address health disparities and reduce cancer burden in local catchment area	USA setting – single center and surrounding catchment area	Community Advisory Board – 3 sites (8-10 members at each site) comprising of survivors, lay caregivers, local cancer advocates, national/regional representatives.	Following poor ratings for Cancer Center Support Grants (address health disparities and reduce cancer burden in catchment area) – a CCC developed a transition team to create a transformative plan for engaging communities to address cancer burden in the catchment area.<br><br>An impact and logic model were developed to serve as a roadmap to monitor progress towards short- and long-term community outreach and engagement goals of the CCC.<br><br>The community outreach and engagement operational strategies draw upon bidirectional partnership, evidence-based practices, and research facilitation to respond to	**System/societal level**<br>Targeted strategies to engage with the community can help address cancer burden, promote health equity and eliminate cancer disparities in the CCC catchment area.

Author, year, and country	Study design / description of source	Aim of study / source	Setting, and type of CCC focus	Population / cancer type focused on	Attributes of CCCs	Impacts
					the critique in the Cancer Center Support Grant application.	
Ringborg 2024, Europe[47]	Meeting report	To describe strategies to decrease inequalities in cancer therapeutics care and prevention, as discussed at the European Academy of Sciences Meeting 23-24	European setting	Nonspecific, cancer in general		CCCs are of critical importance for integrating research innovations to basic and clinical research, and for ensuring state-of-the-art patient care within healthcare systems. International collaborative networks between CCCs are necessary to reach the critical mass of infrastructures and patients for research, and for introducing prevention modalities and new treatments effectively.<br>Data sharing and critical mass are essential for innovative research.<br>Making cancer research infrastructures accessible for all patients, considering the increasing inequalities, requires science policy actions incentivizing research aimed at prevention and cancer therapeutics/care with an increased focus on patients' needs and cost-effective healthcare
Schulmeyer 2024, Europe[48]	Observational – review of medical record	To determine if first opinions at non-CCCs were guideline concordant, in a cohort of people with cancer seeking second opinions at a CCC in Germany	German setting – single CCC	People with urological, gyencological, gastroenterological cancers, and sarcomas (2014-2020) who were seeking a		First opinions in non-CCCs were accordance with the guidelines for 54.5% of patients.<br>The median time taken to form a second opinion was 225 min, and the cancer information service was contacted by patients an average of eight times.<br>Obtaining a second opinion at a CCC gives patients an opportunity to receive a guideline-compliant treatment recommendation and enables them to benefit from newer, individualized therapeutic approaches in clinical trials.<br>Establishing patient-initiated second opinions via central contact points appears to be a feasible option for improving guideline compliance.

Author, year, and country	Study design / description of source	Aim of study / source	Setting, and type of CCC focus	Population / cancer type focused on	Attributes of CCCs	Impacts
				second opinion regarding cancer therapy at a CCC (N=584)		
Trapl 2024, USA[49]	Mixed methods – semi-structured interviews, national survey, and development and utilization of framework	To examine the experiences and perspectives of community engagement by members of a CCC and create and implement a framework to meet the needs of the entire CCC	USA setting – single NCI-CCC	Researchers (n=12 interviews), and CCC members (n=86 survey)	Importance of community engagement, and opportunities for bidirectional engagement recognised by members of the CCC.<br><br>Members of CCC were open to learning new skills, changing approaches, and utilizing services to facilitate engagement and overcome barriers including communication issues, limited awareness of opportunities, and competing priorities.	
Unger 2024, USA[50]	Observational - secondary	To identify a contemporary estimate of enrollment to cancer treatment	USA setting – hospitals accredited by Commission on Cancer	Nonspecific, cancer in general		Participation in cancer studies (including treatment trials, biorepositories, diagnostic trials, economic studies, genetic studies, quality of life studies, and registry studies) was significantly higher at CCCs compared to non-CCCs (e.g., academic

Author, year, and country	Study design / description of source	Aim of study / source	Setting, and type of CCC focus	Population / cancer type focused on	Attributes of CCCs	Impacts
	analysis accreditation data	trials across a diverse set of clinical care facilities in the USA.				comprehensive cancer programs, community cancer programs, and integrated network cancer programs).<br><br>Treatment trial enrollment was 21.6% at NCI-designated comprehensive cancer centers, 5.4% at academic (non–NCI-designated) comprehensive cancer programs, 5.7% at integrated network cancer programs, and 4.1% at community programs. One in five patients (21.9%) participated in one or more cancer clinical research studies.
**Policy / framework documents**						
WHO 2009, Eastern Mediterranean region [51]	Description of strategy for cancer control programs	To promote an integrated approach to cancer control activities and services throughout Eastern Mediterranean region to reduce the incidence of cancer and improve quality of life of those who develop cancer.	Eastern Mediterranean region, accreditation body not discussed	Nonspecific, cancer in general	Important for each country to have one or more specialised cancer centers to provide cancer therapy and coordinate activities with non-specialist hospitals or primary health care providers.<br>Where possible, specialist cancer centers should be comprehensive; able to provide cancer surgery, radiation therapy, medical oncology, paediatric oncology,<br>palliative care, hospital registry, comprehensive imaging, pathology/cytology,<br>medical records, community oncology and other supportive services.<br>CCCs may also have facilities for epidemiology and registration.	

Author, year, and country	Study design / description of source	Aim of study / source	Setting, and type of CCC focus	Population / cancer type focused on	Attributes of CCCs	Impacts
					The CCC should establish links and referral pathways with smaller hospitals and health centers within a regional network.	
Bell 2018, International [52]	Description of framework for CCCs	Cancerpedia is a cohesive framework for CCCs and can be used as a checklist for healthcare professionals looking to develop, scale up or evaluate a cancer center or program.	International (relevant to high-, middle-, and low-income countries). Cancer centers in general.	Nonspecific, cancer in general	**Cancer Control and Care Delivery System**<br>1. Functions; financing, service delivery, resource generation, and stewardship of health systems.<br>2. Components; prevention, screening, diagnosis, treatment, supportive care, palliative and end-of-life care, primary care, supportive care.<br>3. Delivery agents; multiple organisations and individuals who play roles in care delivery.<br>4. Cancer control and care delivery system framework; cancer centers must be integrated into broader system of healthcare and cancer control.<br>**Cancer center includes:**<br>Clinical management, the healthcare team, and patients.<br>Clinical services including medical imaging, laboratory medicine and pathology, surgery, chemotherapy, radiotherapy, supportive care,	

Author, year, and country	Study design / description of source	Aim of study / source	Setting, and type of CCC focus	Population / cancer type focused on	Attributes of CCCs	Impacts
					palliative care, inpatient care, ambulatory/outpatient care, emergency care, pharmacy.<br>Core services/infrastructure including infection prevention and control, physical facilities and support services, equipment and technology, health records, hospital registry, human resources, communication. Governance and quality, research, education, philanthropy, integration with the community.<br>**Beyond the cancer center:**<br>Primary prevention, early detection, and screening programs. Policy and regulations.	
Oberst 2021, Europe [53]	Position paper describing a vision for CCCs	To promote initiatives and policy recommendations that can improve the quality of cancer care received by cancer patients in every country in Europe.	European setting, OECI designated CCCs	Nonspecific, cancer in general	European Union ambitions for CCCs were aligned with the objectives of the EU Cancer Mission and Europe's Beating Cancer Plan's goals including access, regional hospitals and primary care providers need to develop collaborative regional and local networks.<br><br>The EU Network of Comprehensive Cancer Centers should be focussed on specific scientific questions, and result in significant calls for	**Provider level**<br>Integration of research that evaluates the quality of cancer care into clinical care.<br><br>**Organisational level**<br>Optimisation of translational research and research outcomes.<br><br>**System/societal level**<br>Reduce inequalities in diagnosis, treatment, care and access to clinical trials.

Author, year, and country	Study design / description of source	Aim of study / source	Setting, and type of CCC focus	Population / cancer type focused on	Attributes of CCCs	Impacts
					impactful research at both translational, clinical and outcomes research level, leveraging the substantial translational and clinical research work already being undertaken by CCCs.	
Uganda Cancer Institute, Uganda 2022[54]	Article in ASCO International Magazine	To describe the approach to comprehensive cancer control in Uganda	Ugandan setting	Nonspecific, cancer in general	[54, 55]The Uganda Cancer Institute has been mandated by the government to undertake and coordinate the management of cancer and cancer-related diseases in Uganda (a low- to middle-income country where cancer is a major public health problem).<br><br>The Uganda Cancer Institute is focused on: health promotion and cancer prevention services; early detection services; cancer management services (diagnosis, treatment, and palliative care); cancer survivorship services; cancer surveillance and research; and training the next generation of cancer care specialists in Uganda.<br><br>Four regional cancer centres are being established and operationalised by the Uganda Cancer Institute to optimise	

Author, year, and country	Study design / description of source	Aim of study / source	Setting, and type of CCC focus	Population / cancer type focused on	Attributes of CCCs	Impacts
					equitable access to cancer care across the population.<br><br>Uganda Cancer Institute has been designated East Africa Centre of Excellence in Oncology.<br><br>Collaborations with Fred Hutchinson Cancer Research Centre in USA to access medical fellowships in all areas of cancer care.	
**Book chapters**						
Gospodarowicz 2015 (single book chapter), international [56]	Book chapter	To describe the optimal framework for CCCs	International setting, any accreditation body	Nonspecific, cancer in general	All countries should have a cancer control program that includes prevention, screening, diagnosis, treatment, survivorship, palliative and end-of-life care regardless of a country's resource level.<br>A framework for CCCs includes clinical management, clinical services, core services, and comprehensive cancer control.<br>In low- and middle-income countries, CCCs can function as focal points for national cancer control programs.	**Prover level**<br>Training of health care professions.<br><br>**Organisational level**<br>Supporting development of effective health care systems.<br><br>**System/societal level**<br>Development of a national cancer control system and improvement in global health of population.

Author, year, and country	Study design / description of source	Aim of study / source	Setting, and type of CCC focus	Population / cancer type focused on	Attributes of CCCs	Impacts
Aljurf 2022 (editor of book) - 17 chapters included, International [57-73]	Book chapters	To provide guidance on development of comprehensive cancer plan, and to assist cancer centers in upgrading quality in terms of infrastructure, practice standard, policies, and procedures to meet contemporary and highest international standards in a cost-effective and sustainable way.	International setting, any accreditation body	Nonspecific, cancer in general	Core components of CCCs: [64]<br>1. Research and protection of human subjects<br>- basic laboratory research<br>- clinical research in human subjects<br>- translational research<br>- population health research<br>- institutional review boards<br>- clinical research organisations<br>- key programs supporting cancer research<br>2. Clinical care<br>- diagnostics, treatment and follow-up care across multiple disciplines<br>- quality monitoring in cancer care<br>- improving the quality of life of cancer patients through support services (social work, palliative care, navigation, survivorship, education)<br><br>Clinical Services in CCCs: [58, 61, 62, 67-69, 72]<br>- in-patient unit (multidisciplinary team, appropriately designed floor space and rooms, with consideration of environmental demands [i.e., air filtration]).<br>- out-patient care (appropriate clinical team including advanced	**System/societal level**<br>CCCs form the backbone of national cancer control programs and were responsible for developing innovative approaches to cancer prevention, diagnosis, and treatment.

Author, year, and country	Study design / description of source	Aim of study / source	Setting, and type of CCC focus	Population / cancer type focused on	Attributes of CCCs	Impacts
					practice nurses and nurse coordinators; focus on pharmacists, drug administration and preparation; supportive care for complications; ancillary and community services; planning for out-patient chemotherapy and drug delivery; economic advantages of outpatient care; clinical trials; telemedicine; importance of out-patient care during pandemic).<br>- infusion area (appropriate physical space and equipment, appropriate staff, range of on-site services; convenient and potentially cost saving).<br>- establishing radiation therapy facility (population description, needs assessment, equipment [external beam radiotherapy, brachytherapy], considerations for commissioning, room shielding, personnel, quality assurance, lengthy timeline for build, budget).<br>- laboratory / pathology services and blood bank (programs for screening and diagnosis, support in maintaining quality supportive cancer care, pathology support for running stem cell transplant and cellular therapies, support for	

Author, year, and country	Study design / description of source	Aim of study / source	Setting, and type of CCC focus	Population / cancer type focused on	Attributes of CCCs	Impacts
					running cancer clinical trials and research).<br>- pharmacy requirements (appropriate staff, physical spaces, and models of care; importance on a sustainable supply chain [specific issue during pandemic], financially and environmentally).<br>- transplantation and cellular therapies (require appropriate structure and infrastructure, appropriate staff, quality management, and data and research; recent advances in cellular therapy).<br><br>Workforce in CCCs: [57, 63]<br>- oncology nursing care (specialised roles in in-patient unit, ambulatory setting, survivorship, palliative care and hospice, as well as roles outside of clinical setting)<br>- administrative services (strategic planning for future, space and facilities planning and program development, financial management, budgeting, performance management, regulatory and accreditation standards, human resources management, access services,	

Author, year, and country	Study design / description of source	Aim of study / source	Setting, and type of CCC focus	Population / cancer type focused on	Attributes of CCCs	Impacts
					scheduling, insurance verification and medical record collection, referral management).<br><br>Supportive care and patient resources in CCCs: [60, 71, 73]<br>- psychosocial and patient support services (appropriate services and models of care for physical and psychological distress, community outreach of services, resource-limited settings should focus on greatest need when providing support services).<br>- starting a palliative care service (requires long-term planning, choice of palliative care delivery model/s [inpatient consulting, inpatient admitting, outpatient clinic, community based], successful integration with clinical care requires education of staff, important to monitor quality, research framework important for a sustainable service).<br>- patient resources (treatment education, support services [financial, palliative care, social work, case managers, nutritional therapists, religious and spiritual]).	

Author, year, and country	Study design / description of source	Aim of study / source	Setting, and type of CCC focus	Population / cancer type focused on	Attributes of CCCs	Impacts
					Quality measures in CCCs: [59]<br>- designing quality from ground up (three dimensions of quality should be measured; structure, process, and outcome).<br><br>Research and translation in CCCs: [66]<br>- data unit, translational research, registries (CCC should have a research data unit, focus on translational research, and disease specific registries).<br><br>Education and training in CCCs: [70]<br>- education and training (appropriate training for multidisciplinary health care professionals, continued medical education [ground rounds, board certification, development of new cancer-specific programs]).<br><br>Challenges/opportunities for sites with limited resources: [65]<br>- medical services, data and infrastructure (need for effective cancer registries, lack of connectivity, research infrastructure, tumor boards and multispecialty	

Author, year, and country	Study design / description of source	Aim of study / source	Setting, and type of CCC focus	Population / cancer type focused on	Attributes of CCCs	Impacts
					care), human resources (unavailability of specialised services [radiotherapy and stem cell transplantation], education infrastructure), quality management and access to care (sustainability and consistency, access to cancer care, influence of political activities (refugee crises and internal displacement), locally successful / appropriate models of care, drug approvals and shortages, safe and effective institutional blood bank, applying technological advancement sin oncology, public health crises.	
**Thesis**						
Rajan 2015, Europe [74]	Doctoral thesis including the following: 1. Survey 2. Interviews	Main aim: to present an assessment framework to identify and designate excellent European CCCs in translational research.<br><br>Additional aims: i) to explore the	European setting, OECI designated CCCs	Nonspecific, cancer in general	Proposed framework for assessing excellence in translational research in CCCs (6 domains): i) organisational policies and strategies; ii) people management; iii) research and infrastructure components; iv) clinical trial management, v) internationally recognised excellence; vi) financial expertise.<br>Assessing excellence of translational research is essential to improving its performance so that	

Author, year, and country	Study design / description of source	Aim of study / source	Setting, and type of CCC focus	Population / cancer type focused on	Attributes of CCCs	Impacts
	3. Literature review<br>4. Development of framework<br>5. Surveys<br>6. Pilot	benefits of participating in accreditation process; ii) to explore benefits associated with CCCs.			the time lag in binging results of research to practice will be reduced. Assessment needs to be flexible because excellent translational research can be found in areas that are outside the scope of typical definitions of translational research.	
**Websites**						
Australian Comprehensive Cancer Network, Australia[75]	Website for Australian Comprehensive Cancer Network	To outline a newly established national Comprehensive Cancer Network	Australian setting, no accreditation and designation system	Nonspecific, cancer in general	Australian Comprehensive Cancer Network aims to address disparities in outcomes across Australia through an integrated national network that connects cancer services.<br><br>Multidisciplinary clinicians across public and private health systems will be virtually linked to CCCs.<br><br>CCCs will be centers of excellence, linked together and across the network.	Health services will have the opportunity to connect with CCCs (and other centers of excellence) that can provide specialist knowledge, clinical expertise, and capability.<br><br>Ensure that all patients throughout Australia will receive care as close to home as safely possible, particularly those in regional and rural locations.

Author, year, and country	Study design / description of source	Aim of study / source	Setting, and type of CCC focus	Population / cancer type focused on	Attributes of CCCs	Impacts
Czech Cancer Center Network, Czech Republic [76]	Website for National Cancer Control Program	To define the criteria of the status of CCC	Czech setting, CCC status determined by Czech Cancer Center Network	Nonspecific, cancer in general	CCC status must include:<br>-Staff that medical oncology and radiation oncology specialties have at their disposal<br>- radiotherapy equipment, and equipment for systemic therapy<br>- interdisciplinary cooperation, coordination of professional help for other facilities, education and research, specialist training<br>- requirements for diagnostic methods, medical oncology, radiation oncology<br>- surgical care specifically focused on cancer patients<br>- supportive and palliative care Maintenance of documents on cancer patients<br>- follow-up care of cancer patients<br>- clinical trials and implementation of new procedures<br>- active participation in the organisation of cancer care in respective region	
CraNE, European Union members states[77]	Website for European Network of Compr	To outline the CraNE Joint Action that aims to establish a European Union network of recognised CCCs	European setting, new designation system to developed	Nonspecific, cancer in general	The new European network of CCCs will:<br>- develop a governance model for the European Union Network of CCCs	**Organization level**<br>Network of CCCs developed practical instruments to govern oncological care successfully (i.e., specific patient pathways, development of quality indicators, development of monitoring frameworks for development of CCCs).

Author, year, and country	Study design / description of source	Aim of study / source	Setting, and type of CCC focus	Population / cancer type focused on	Attributes of CCCs	Impacts
	ehensive Cancer Centers	in every member state, to support Europe's Beating Cancer Plan to address disparities in cancer care across the European Union.			- guarantee a high standard of care and research in each CCC through a continuous update mechanism<br>- foster collaboration within the Network and with other European initiatives.<br><br>When developing new a designation system for CCCs, experience from current systems must be built upon. While clear consistent selection and evaluation criteria is necessary, a flexible approach is needed that considers the regional context of various countries.	**System/societal level**<br>CCCs and network of CCCs will be the foundation to reach the goal of the Europe's Beating Cancer Plan to ensure high standards in cancer care and reduce inequalities in care.<br><br>Networking of CCCs is a strategy to reduce inequalities in care, strengthen the quality of research, and integrate clinical care and research.
National Cancer Institute, USA [78]	Website for National Cancer Institute	The NCI Cancer Centers Program is one of the anchors of the nation's cancer research effort. NCI recognizes centers nationally that 'meet rigorous standards for transdisciplinary, state-of-the-art research focused on developing new and better	USA setting, NCI-CCCs and CCs	Nonspecific, cancer in general	**NCI CCCs** demonstrate reasonable depth and breadth of cancer research activities in each of three major areas: basic laboratory; clinical; and prevention, control, population-based science, as demonstrated in the formal Research Programs'.<br><br>NCI CCCs also have substantial transdisciplinary research across these three areas.<br><br>NCI CCCs serve their catchment area as well as the broader population through research and	

Author, year, and country	Study design / description of source	Aim of study / source	Setting, and type of CCC focus	Population / cancer type focused on	Attributes of CCCs	Impacts
		approaches to preventing, diagnosing, and treating cancer'.			cancer control activities – demonstrated in Community Outreach and Engagement.<br><br>NCI CCCs integrate training and education of biomedical researchers and community health care professionals into scientific program, demonstrated by the Cancer Research Training and Education Coordination component.<br><br>NCI CCCs must be strong in all four areas listed above. 'Strengths in some areas cannot mitigate weaknesses in one or more other areas'.<br><br>Six essential elements include: Physical space, organizational capabilities, transdisciplinary collaboration and coordination, cancer focus, institutional commitment, and a center director.	

Author, year, and country	Study design / description of source	Aim of study / source	Setting, and type of CCC focus	Population / cancer type focused on	Attributes of CCCs	Impacts
National Comprehensive Cancer Network, USA [79]	Website for National Comprehensive Cancer Network	Alliance of leading cancer centers devoted to patient care, research, and education. Dedicated to improving and facilitating quality, effective, equitable, and accessible cancer care so all patients can live better lives.	USA, NCI-CCCs and CCs	Leading NCI-CCCs, cancer in general	Characteristics of the centers in alliance include:<br>Leadership and expertise in clinical professionals at member institutions; pioneer multidisciplinary approaches to patient care; conduct innovative research.	**Provider level**<br>Attract world-renowned experts from member institutions diagnose and treat patients with broad spectrum of cancers and rare and complex cancers.<br>Patient advocates, support, education.<br>Offers access to expert physicians, superior treatment, and quality and safety initiatives.<br><br>**Organisational level**<br>Guideline development, research, engagement with stakeholders, promoting effective policies.<br><br>**System/societal level**<br>Define and advance high-quality cancer care.
National Comprehensive Cancer Network Global Program, USA[80]	Website for National Comprehensive Cancer Network Global Program	NCCN Global program seeks to facilitate quality, effective, equitable, and accessible cancer care through all regions of the world	International setting	Nonspecific, cancer in general	NCCN Global Program has developed strategic global initiatives to ensure awareness, accessibility, and relevancy of NCCN resources.<br><br>Programs facilitates translation of NCCN clinician and patient guidelines and clinical tools, adaptations/harmonization of clinical guidelines, and other global support initiatives.<br><br>NCCN Global Collaborations now exists in Africa, Asia, Latin America, Europe, Middle East and North Africa, and Worldwide.	**System/societal level**<br>Increase global access to high quality, high value cancer care.

Author, year, and country	Study design / description of source	Aim of study / source	Setting, and type of CCC focus	Population / cancer type focused on	Attributes of CCCs	Impacts
Organisation of European Cancer Institute, Europe [81]	Website for Organisation of European Cancer Institute	The OECI accreditation program has the following goals: to provide cancer patients equal access to high quality of cancer care and overcome the current differences in access to diagnostics, treatment, and therapeutic options that patients' cancer experience; to help European cancer centers implement a quality system for oncology care; to foster and accelerate improvements in translational and clinical cancer research.	European setting, OECI designated CCCs	Nonspecific, cancer in general	OECI cancer centers (including CCCs) must have: i) organisational entity with clear governance; ii) direct provision of an extensive variety of high-quality cancer diagnostics and care tailored to the patient's needs; iii) culture of learning and improving professional organisational quality of care.<br>In addition, CCCs must also demonstrate: i) high level of infrastructure, expertise and innovation in cancer research, especially in translational and clinical research, but also in many cases including basic science; ii) either strong University and Research Institute links, or a University partnership as part of the Comprehensive Cancer Center; iii) extensive international networking.<br>Specific criteria for CCC designation included:<br>Planned annual budget for oncology care and research. Careful records of numbers of newly treated cancer patients per year, inpatient and outpatient beds/chairs, prospective studies open in year, percentage of patients in clinical trials, number of	**Provider level**<br>Accreditation program is contributing to improvements in development of consistent procedures for multidisciplinary team.<br>Quality and risk management (focused on cancer care and new technologies.<br>Patient empowerment, including education and engagement in designing new services and research studies.<br><br>**Organisational level**<br>Accreditation program is contributing to improvements in: Integration of research into clinical care, and efficient use of resources.<br>Data collection and analysis of different types of cancer, treatment, and outcomes.<br>Education, training, and an increase in the number of clinical trials and proportion of people enrolled into trial.<br><br>**Systems level**<br>Accreditation program is contributing to improvements in: Creation of international scientific advisory boards.

Author, year, and country	Study design / description of source	Aim of study / source	Setting, and type of CCC focus	Population / cancer type focused on	Attributes of CCCs	Impacts
					oncology related peer reviewed pubs per year.<br>Centers must cover radiotherapy, surgery and medical oncology.	
**White papers**						
McArthur 2022, Australia [55]	Pre-budget submission	Outline priority areas in cancer research, treatment and care for the CCC.	Single CCC in Australia, no accreditation system discussed	Nonspecific, cancer in general	The VCCC aims to be recognised as a global center of excellence in cancer research<br>and evidence-based cancer care, with a key focus on reducing inequities in cancer outcomes.	**Provider level**<br>Successful delivery of achievements including increased participating in clinical trials in metropolitan and regional areas; establishment of innovative, collaborative immunotherapy program; development of Master of Cancer Sciences through University of Melbourne.<br><br>**Organisational level**<br>Rapid translation of cancer research and development of workforce.<br>Enhance/networks and facilitates the development and application of better cancer care across and between member organisations.<br><br>**System/societal level**<br>Delivery of integrated, world-leading program of cancer research, education and clinical care in metropolitan, regional and rural parts of Victoria.
German Cancer Aid 2023, Germany[82]	Application guidelines 'Oncol	To describe the application process and criteria for support from the	German setting, CCC certification and	Nonspecific, cancer in general	Three equally important areas for 'Oncology Centers of Excellence':<br>1. translational oncology, access to innovation, clinical trials	

Author, year, and country	Study design / description of source	Aim of study / source	Setting, and type of CCC focus	Population / cancer type focused on	Attributes of CCCs	Impacts
	ogy Centers of Excellence'	German Cancer Aid based on designation as 'Oncology Center of Excellence' (CCC)	designation system		2. outreach, regional cancer network<br>Basic information / size of center<br>3. multidisciplinary care<br><br>Criteria includes the following:<br>- characteristics and size of center<br>- leadership and organisational structure<br>- research activity and translational oncology<br>- research infrastructure<br>- access to innovation (molecular diagnostics, precision medicine, immunotherapy, early clinical trials)<br>- clinical trials activity<br>- clinical trials infrastructure<br>- regional network and outreach activity<br>- community outreach<br>- multidisciplinary care<br>- tumor documentation, clinical cancer registry, and information technology<br>- palliative care<br>- psychosocial care and self-help groups<br>- patient engagement and involvement<br>- training programs	

CAM: complementary and alternative medicine; CCC: comprehensive cancer center; CC: clinical/cancer center; EACS: European Academy of Cancer Sciences; ESMO; INDOX: INDia-Oxford; DKDT: Deutsche Zentren Der Gesundheitsforschung (German Cancer Research Center); IRCCS: Instituti di Ricovero e Cura a Carattere Scientifico; N/A: not applicable; NCCN: National Comprehensive Cancer Network; NCI: National Cancer Institute; OECI: Organization of European Cancer Institutes; vs: versus; VCCC; Victorian Comprehensive Cancer Center.

Supplementary File S4. Narrative summary of key attributes and impacts of CCCs

Clinical service provision

The comprehensiveness of clinical services at CCCs was discussed in 27 international sources [2-4, 7-9, 11-14, 16, 17, 19, 22-25, 29, 30, 33, 36, 42, 52, 74, 83, 84]. Core services included medical imaging, laboratory medicine and pathology, transplantation and cellular therapies, innovative and experimental services, surgery, chemotherapy, radiotherapy, supportive care, palliative care, inpatient care, ambulatory/outpatient care, emergency care, pharmacy, and data and research unit [3, 4, 7, 8, 11, 12, 14, 25, 29, 52, 83]. A variety of core infrastructure to support comprehensive clinical service provision at CCCs, including infection prevention and control, physical facilities and support services, equipment and technology, health records, hospital registry and human resources, was reported in 6 sources from various countries [3, 4, 13, 29, 52].

A diverse range of multidisciplinary clinical and non-clinical roles were highlighted as crucial to provide high-quality comprehensive services including specialized oncology nurses, allied health (physiotherapy, speech pathology, social work, occupational therapy, dieticians), care coordinators and navigators, pharmacists, medical staff across various specialties, radiation therapists, scientists (including pathology, blood bank, cellular therapy and stem cell transplant technicians), research staff (including ethics administrators), administrators (managers, executives, insurance coverage), and patient support staff [58, 61, 62, 67-69, 72]. Consumer engagement to inform clinical service provision was reported as an important component of high-quality person-centered service provision in four studies [15, 21][13, 74]. Six sources discussed the role of CCCs in providing high quality clinical services, and complex care throughout the cancer trajectory including prevention, diagnostics, treatment, follow-up, end-of-life care [3, 4, 8, 19, 29, 83]. Cancer prevention services led by CCCs were reported to be important to improve population health, respond to shortage of healthcare resources, and reduce carbon emissions from cancer care [45].

Primary data from OECI designated and NCI designated CCCs showed, however, that availability of these services varied between CCCs [55, 84]. The impact of COVID-19 was noted as key challenge for CCCs, with considerable numbers of hidden or delayed cancer diagnoses resulting from reduced medical care during the pandemic [55, 84]. In addition to the core cancer services listed above, CCCs were reported to provide innovative and experimental services such as ovarian cryopreservation and new diagnostic methods in 3 studies [7, 8, 25]. Managing quality-of-life through various supportive care and integrated and complementary care services was discussed by 11 sources including both primary research and opinion pieces [2-4, 7, 9, 17, 19, 22, 24, 33, 83]. A systematic review of NCI designated CCCs and community hospitals reported that the availability of both integrative medicine and integrative care was significantly higher in CCCs than other community hospitals [33]; primary studies have shown that the availability of these services have increased significantly over time [16, 22]. In four sources, use of treatment pathways was reported to lead to excellence in patient-centered care delivery, but was dependent on availability of services at individual CCCs [8, 13, 29, 30]. Furthermore, technology-backed care delivery, digitally enabled hospitals and integrated healthcare information systems were seen as important aspects of clinical care delivery at CCCs that could lead to improved patient safety, information sharing, and patient wait times [13, 29].

Location of CCC services was discussed in 11 sources, advising that CCCs should ideally be in one visible location or under one roof, as a 'one stop shop' [2-4, 11, 12, 14, 15, 25, 29, 52, 83]. Location of a CCC in one location was perceived to improve patient outcomes and quality

of care by providing a more complete approach to multidisciplinary care through integration of disciplines, collaborations, and accessibility to appropriate services [12, 13, 25]. Further impacts identified in both primary studies and opinion pieces included increased clinical efficiencies and reduced costs (via Integrated Practice Units within CCCs) [29] and the ability to deliver more complex treatments [12]. Access to a critical mass of patients for research and resources [30, 36, 84] and swift adoption of novel therapies and other innovations with demonstrated impacts into clinical guidelines and practice were also identified as potential benefits for CCCs in one location [23, 36]. However, an alternative model of CCC was described in Australia [55] and Africa [54], where comprehensive cancer services were provided across various sites.

Research, data, and innovation

The leading role that CCCs play in research, data, and innovation was presented in 29 sources from Europe [3, 5, 11, 13, 15, 21, 23, 25-27, 29-31, 36, 38, 53, 74, 81, 82, 84], the USA [28, 35, 37, 85], internationally [52, 83], India and UK [6], Australia [55], and Singapore [4]. CCCs were perceived to provide a collaborative stimulating environment for basic researchers to interact closely with clinicians from a variety of in clinical specialities,[23] and have access to a large numbers of diverse patient groups [13, 29, 83]. Research and innovation were core criteria for CCCs that distinguished them from other cancer centers [81, 82, 85]. Twelve sources acknowledged the variation in clinical specialties as a strength in pioneering innovation through high-quality translational ‘bench to bedside’ research [3, 4, 11, 25, 26, 29, 30, 36-38, 52, 83]. To support this, a secondary analysis of OECI accreditation data identified that OECI designated CCCs had significantly more clinical trials open to recruitment, higher numbers of recruitment per index year, and more high-impact publications than CCs in the same region [36]. Analysis of academic outputs of the surgical faculties in NCI designated CCCs showed that surgeons in these hospitals had significantly higher academic outputs, qualifications and a history of higher NIH funding compared to CCs in the US [28].

The use of large sets of outcome data to drive research,[29] national cancer data strategies and data linkages, to speed up the pace of translation of research into policy and practice [55] was highlighted as an important function of CCCs. Real-world data on large patient cohorts, followed for many years in CCCs, can facilitate long-term tracking of outcomes via data lakes/warehouses [29]. This data can be shared to inform cancer prevention, detection, treatment, and supportive care efforts [55]. Population level data registries were believed to be vital to research on factors associated with cancer development in coming years, providing critical link between pre-clinical and clinical research [29]. The capability to translate research from basic science to clinical implementation quickly through the ability to rapidly recruit research participants was presented as an important attribute of CCCs in six sources [21, 23, 25, 29, 36, 84]. This capability was reported to lead to clinically relevant [23] and cost-effective, home-grown innovations [74], and supports the development of streamlined treatment pathways [35], evidenced-based clinical guidelines [23, 29-31, 53], and cancer registries [13]. Of note, 24 of the 32 (75%) studies included in the SR used data from long-standing state or national population-level registries to compare outcomes between CCCs and non-CCC [86-109].

Support and infrastructure for high-quality research activity within CCCs was reported in 11 sources. These included protected research time for clinician researchers [84], uniform quality standards for research [6], and dedicated state-of-the-art technology and clinical trials units to support research both within CCCs and collaboratively with other organisations [13, 26, 27,

29, 30, 74, 83, 84]. The establishment of biobanks, and access to longitudinal data was also seen as an attribute of CCCs that leads to major clinical advances.[5, 21] Beyond single CCCs, networks of CCCs have been shown to create structures that support less experienced CCCs [6, 26], address key areas of cancer research and facilitate international collaborations for clinical trials leading to greater accessibility to clinical trials for more patients [15, 23, 36], particularly patients with rare cancers [15], and people living in lower income countries [6]. Furthermore, six sources identified that strengthening community outreach through sharing research data and resources with other healthcare organizations was a feature of CCCs research activity [26, 27, 35, 74, 84]. Finally, three sources also identified that CCCs had a strong focus on consumer and community engagement in their research [13, 74]·[21], which was particularly important to support knowledge translation [21].

Education and clinical support

The role of CCCs in teaching, and clinical support was highlighted in 18 sources [4-6, 21, 25, 29, 36, 38, 39, 52, 55, 56, 83]. Ten of these sources identified that CCCs leadership in education and clinical support involved comprehensive, interdisciplinary, high-quality education, training and mentoring of healthcare professionals and all staff within organisation in both clinical care and research [5, 6, 21, 25, 29, 36, 52, 55, 56, 83]. These education and teaching programs manifest through the development of standard operating procedures and best practice guidelines for staff [25], alignment of research, care and education,[38] and the delivery of post graduate cancer programs and medical training programs [4, 55]. Outreach teaching and mentorship to outside organisations were reported in the form of tumor boards, online resources and toolkits and conferences [25, 39]. Public education forums [4] and facilitated knowledge sharing between academic CCCs and community hospitals using web-based question and answer platforms [39] were also seen as important attributes to support high-quality patient care in hospitals outside CCCs.

Impacts of high-quality education and training programs were discussed in six sources [2, 5, 12, 29, 42, 55] Stated impacts included high levels of staff satisfaction [2, 42], greater teaching and collaboration outcomes due to staff being located in one building [42], increased support for the development and implementation of national health plans by upskilling staff [5] and timely information exchange regarding provision of complex clinical care [12]. These programs were shown to support the development of the CCC and regional cancer workforce through completion of post graduate studies [55] and career advancement opportunities for staff [29].

Leadership and networks

The leadership role of CCCs, particularly within networks and alliances, was explored in 29 sources in European [3, 5, 8, 12, 15, 18, 21, 25, 26, 29, 30, 36, 38, 51, 77, 84], USA [39, 42, 43, 79], Indian and UK [6], Australian [55, 75], and international settings [52, 56]. Two main types of networks were discussed including: 1) networks of geographically dispersed CCCs across a nation or continent; and 2) networks between CCCs and community providers and smaller/non-specialised hospitals to serve a geographical region (most commonly discussed in the literature). An Australian and Ugandan approach to delivery of comprehensive cancer care via networks of CCC infrastructure and services was discussed.[54, 75] Networks of CCCs were described in European [3, 10, 26, 77], German [82], Italian [12, 15], and USA setting and were focused on co-operation and collaboration to strengthen research and clinical care [6, 15, 18, 38, 52, 79]. Impacts of networks of CCCs included enhanced international collaboration with world-renowned experts, guideline development, enhancing research capacity across care continuum, training of staff, and linking with cancer patient organisations [6, 10, 15, 18, 38, 79]. The only source set in a middle-income country reported a network of

CCCs across India, supported by an alliance with a leading university in the UK to conduct high-quality, locally relevant research [6].

Five sources discussed the important leadership role that networks or standalone CCCs have in community outreach and support across catchment areas to support delivery of best practice care [25, 29, 39, 51, 52]. Even in countries with good availability of CCCs, most people with a cancer diagnosis will not receive care in a CCC, and it may be appropriate to decentralize some cancer care services [25]. Networking between a CCC and the local community (healthcare providers, patients/families, and advocacy groups) was reported to be essential to provide equitable care to the community [25, 29, 39, 51, 52]. Support for community oncologists was noted to be an important function of local CCC networks [39]. The leadership role that CCCs played during the COVID-19 pandemic in supporting people with cancer to access timely, safe, appropriate care was also noted [55].

Staff within CCCs were reported to be leaders in their fields with secondary analysis of OECI accreditation data reported staff in CCCs more consistently are leaders in international Scientific Advisory Boards [36]. In networks or as standalone facilities, CCCs were reported to have significant influence and impact on cancer related issues and national cancer strategies [3, 29, 38, 84] including across primary prevention, early detection, and screening programs [52]. The leadership role of CCCs in shaping national cancer control programs was reported as particularly important in low- and middle-income countries [56]. Included sources also described the leadership role staff in CCCs played in the development of clinical guidelines [5, 77]. Perceived high level impacts associated with the leadership and networks of CCCs were reported to be improved cancer control and healthcare systems, improved population health, and reduced mortality and morbidity [3, 56, 83]. Effective leadership within CCCs was also noted as important [36], to enable clear vision and articulated intention [21], and was part of accreditation criteria in the USA and Europe [82, 85]. Gender composition, or inclusion of people with lived experience in leadership teams was not discussed in any sources.

Health equity and inclusiveness

Issues surrounding health equity related to CCCs at an international, national and local level were discussed in 14 sources [15, 20, 23, 25, 29, 34, 35, 37, 38, 40, 43, 51, 53, 56]. In European sources [38, 77], CCCs were reported as pivotal to delivering the Cancer Mission, and enabling Europe's Beating Cancer Plan, which focuses largely on inclusiveness and health equity across all European member states, by working towards equitable access to comprehensive cancer care [110]. In international sources, it was recommended that all countries should have at least one specialised cancer center (where possible a CCC) however, the challenges associated with low- and middle-income countries achieving this was noted [51, 56].

Two US studies highlighted issues with equitable access to care for vulnerable populations at CCCs [20, 40]. A secret shopper method found equitable acceptance for care, but longer wait times to first appointment, and gate-keeping behaviors from administrative staff for callers posing as patients with Medicaid insurance compared to those with private insurance [20]. A retrospective cohort study found African American patients had longer wait times between diagnosis and presentation to a NCI-CCC, and were more likely to undergo initial care at a non-CCC, compared to White people [40]. In the same study, Hispanic people were more likely to present with advanced stage disease at an NCI-CCC compared to non-Hispanic [40].

Sources reported that core business of CCCs was to reduce inequities in diagnosis, treatment, care, and access to clinical trials in metropolitan, regional, and rural areas [53, 55]. In the USA, sources reported that NCI designated CCCs were called to adopt and resource an explicit health equity approach, driven by local data and health equity policies, to address increased

quality of care and clinical trial enrolment of minority populations [34, 35]. In Europe and the USA, CCCs were reported to be responsible for outreach with smaller hospitals and networking across catchment areas to address health equity [29, 38, 51] and were incentivised to do this via through earmarked funding [43]. In Australia (a country with a dispersed population), a CCC was reported to provide clinical support across regional and rural areas, supporting equity in access to high-quality cancer care [55].

In the only study set in a middle-income country, Ali and colleagues [6] described a network of CCCs across India that had partnered with a leading university in the UK to enhance local research capacity and expertise to address local priorities. Research within networks of CCCs were reported as avenues to improve health equity through collaboration [38], leading to increased availability of clinically trials and ability to focus on rare types of cancers [15]. One source described the Global Program, which develops strategic global initiatives to ensure awareness, accessibility, and relevancy of NCCN resources [80].

Accountability and governance

Fourteen sources set in the USA, European countries, UK, or relating to any country, focused on accountability of CCCs provided via accreditation and designation programs [3, 12, 14, 16, 27, 53, 74, 76, 77, 81-85]. Supplementary File S5 provides a high-level summary of the three current CCC accreditation and designation programs; NCI, OECI, and German Cancer Aid. Each of these programs used a tiered approach, where facilities were granted designation based on their purpose and function, and if they met prescribed criterion. Accreditation criteria outlines the essential characteristics and standards that were subjectively assessed, distinguishing CCCs from other types of cancer centers, allowing each center to use the title 'NCI' or 'OECI' CCC. Research activity and output was a key criterion that separated CCCs from CCs. In Germany and the USA, designated CCCs were able to access 'earmarked' government research funding [82, 85]. In the USA and European countries, accreditation and designation programs have been in place for many years, while globally many countries do not have formal systems that aim to promote accountability [4, 55].

Sources focused on accreditation and designation programs mostly consisted of opinion/commentary publications and websites, and described a range of perceived impacts including defining and advancing high-quality performance in patient-centered care, research, data, and innovation, staff training and education, and enhanced health equity [81, 82, 85]. Designation of CCCs was also reported to enable allocation of research funding, and establishment of international scientific boards of clinicians working within CCCs [81, 82, 85]. In the European members states, the value of accreditation and designation programs is reflected in a large body of work currently underway to establish a new accreditation and designation system, and network of CCCs [77].

Two observational studies reported on secondary analyses of accreditation data [11, 36]. Kerloesser and colleagues [36] reported key differences between CCCs and CCs in organisational structure and governance - specifically around superior corporate strategic planning, quality of patient outcome data, diagnostic trends reported by centers, and evaluation of the effect of improvement actions in CCCs. The same study also reported CCCs had better compliance compared to CCs with OECI quality standards for leadership and management and research, innovation, and development, and were supported by significantly larger budgets for oncology care. In other areas of accreditation criteria, key differences were reported between CCCs and CCs regarding greater number of oncology patients cared for, research outputs, clinical trials open for recruitment, patients recruited to clinical trials, conduct of translational research, and scientific knowledge transfer programs [36].

Saghatchian and colleagues [11] reported that five of the first 10 centers to apply for OECI designated CCC designation status failed; three relating to research shortcomings (i.e., lack of publications or integration between laboratories); one due to research and care limitations

(i.e., lack of harmonisation between patients and quality policy guidelines); and one due to care issues (i.e., lack of identified structure for cancer management). These differences were reported to separate CCCs from CCs in accreditation and designation programs [11].

Four sources [3, 12, 27, 77] discussed the importance of national bodies or organisations overseeing CCCs and networks of CCCs. This type of governance was reported to support the establishment of CCCs, networks of CCC, accreditation and designation programs, address health equity, and link into national or regional cancer plans.

Supplementary file reference list

1. Tricco, A.C., et al., *PRISMA extension for scoping reviews (PRISMA-ScR): checklist and explanation.* Annals of Internal Medicine, 2018. **169**(7): p. 467-473.
2. Ford, J.L., *Mind, body and spirit: Comprehensive cancer centers emphasize treating the whole person.* ASRT (American Society of Radiologic Technologists) Scanner, 2004. **36**(11): p. 8-11.
3. Ringborg, U., et al., *Managing cancer in the EU: The Organisation of European Cancer Institutes (OECI).* Eur J Cancer, 2008. **44**(6): p. 772-3.
4. Soo, K.C., *Role of comprehensive cancer centres during economic and disease transition: National Cancer Centre, Singapore--a case study.* Lancet Oncology, 2008. **9 1077-4114 (Print)**(8): p. 796-802.
5. Pelagio, G., D. Pistillo, and M. Mottolese, *Minimum biobanking requirements: issues in a comprehensive cancer center biobank.* Biopreserv Biobank, 2011. **9**(2): p. 141-8.
6. Ali, R., A. Finlayson, and Indox Cancer Research Network, *Building capacity for clinical research in developing countries: the INDOX Cancer Research Network experience.* Glob Health Action, 2012. **5**.
7. Clayman, M.L., et al., *Oncofertility resources at NCI-designated comprehensive cancer centers.* J Natl Compr Canc Netw, 2013. **11**(12): p. 1504-9.
8. Deriu, P.L., et al., *Accreditation for excellence of cancer research institutes: Recommendations from the Italian Network of comprehensive cancer centers.* Tumori, 2013. **99**(6): p. 293e-8e.
9. Van Harten, W.H., A. Paradiso, and M.M. Le Beau, *The role of comprehensive cancer centers in survivorship care.* Cancer, 2013. **119**: p. 2200-2201.
10. Eggermont, A.M., et al., *Cancer Core Europe: a consortium to address the cancer care-cancer research continuum challenge.* Eur J Cancer, 2014. **50**(16): p. 2745-6.
11. Saghatchian, M., et al., *Pioneering quality assessment in European cancer centers: A data analysis of the organization for European cancer institutes accreditation and designation program.* J Oncol Pract, 2014. **10**(5): p. e342-9.
12. Ancarani, V., et al., *The Comprehensive Cancer Care Network of Romagna: The opportunities generated by the OECI accreditation program.* Tumori, 2015. **101 Suppl 1**: p. S55-9.
13. Canitano, S., et al., *The Regina Elena National Cancer Institute process of accreditation according to the standards of the Organisation of European Cancer Institutes.* Tumori, 2015. **101 Suppl 1**: p. S51-4.
14. Da Pieve, L., et al., *The OECI model: The CRO Aviano experience.* Tumori, 2015. **101 Suppl 1**: p. S10-3.
15. De Paoli, P., et al., *Alliance Against Cancer, the network of Italian cancer centers bridging research and care.* J Transl Med, 2015. **13**: p. 360.
16. Hammer, S.L., et al., *Seventeen years of progress for supportive care services: A resurvey of National Cancer Institute-designated comprehensive cancer centers.* Palliat Support Care, 2015. **13**(4): p. 917-25.
17. Platek, M.E., et al., *Availability of outpatient clinical nutrition services for patients with cancer undergoing treatment at comprehensive cancer centers.* Journal of Oncology Practice, 2015. **11**(1): p. 1-5.
18. Ricciardi, W., *Health systems, quality of health care, and translational cancer research: the role of the Istituto Superiore Sanità - Rome.* Tumori, 2015. **101 Suppl 1**: p. S67-8.
19. Berendt, J., et al., *Integrating palliative care into comprehensive cancer centers: Consensus-based development of best practice recommendations.* Oncologist, 2016. **21**(10): p. 1241-1249.

20. Hamlyn, G.S., et al., *Accessibility and barriers to oncology appointments at 40 National Cancer Institute–designated comprehensive cancer centers: results of a mystery shopper project.* Journal of oncology practice, 2016. **12**(10): p. e884-e900.
21. Rajan, A., et al., *Excellent translational research in oncology: A journey towards novel and more effective anti-cancer therapies.* Mol Oncol, 2016. **10**(5): p. 645-51.
22. Yun, H., L. Sun, and J.J. Mao, *Growth of integrative medicine at leading cancer centers between 2009 and 2016: A systematic analysis of NCI-designated comprehensive Cancer Center Websites.* J Natl Cancer Inst Monogr, 2017. **2017**(52).
23. Adami, H.O., et al., *European Academy of Cancer Sciences: Position paper.* Mol Oncol, 2018. **12**(11): p. 1829-1837.
24. Rolland, B. and J. Eschler, *Searching for survivor-specific services at NCI-designated comprehensive cancer centers: A qualitative assessment.* J Natl Compr Canc Netw, 2018. **16**(7): p. 839-844.
25. Brandts, C.H., *Innovating the outreach of comprehensive cancer centers.* Mol Oncol, 2019. **13**(3): p. 619-623.
26. Eggermont, A.M.M., et al., *Cancer Core Europe: A translational research infrastructure for a European mission on cancer.* Mol Oncol, 2019. **13**(3): p. 521-527.
27. Joos, S., et al., *German Cancer Consortium (DKTK): A national consortium for translational cancer research.* Mol Oncol, 2019. **13**(3): p. 535-542.
28. Kim, B.J., et al., *National Cancer Institute centers and Society of Surgical Oncology Cancer Research synergy.* J Surg Res, 2019. **236**: p. 92-100.
29. Oberst, S., *Bridging research and clinical care: The comprehensive cancer centre.* Mol Oncol, 2019. **13**(3): p. 614-618.
30. Berns, A., et al., *Towards a cancer mission in Horizon Europe: Recommendations.* Mol Oncol, 2020. **14**(8): p. 1589-1615.
31. Gahr, S., et al., *Implementation of best practice recommendations for palliative care in German comprehensive cancer centers.* Oncologist, 2020. **25**(2): p. e259-e265.
32. Pasick, R.J., et al., *Unlocking the vault: Can 2nd opinions by comprehensive cancer center breast oncologistsimprove treatment quality for African Americans?* 2020. **29**(6 SUPPL 2).
33. Desai, K., et al., *Availability of integrative medicine therapies at National Cancer Institute-designated comprehensive cancer centers and community hospitals.* J Altern Complement Med, 2021. **27**(11): p. 1011-1013.
34. Doykos, P.M., et al., *Special convening and listening session on health equity and community outreach and engagement at National Cancer Institute-designated comprehensive cancer centers.* Health Equity, 2021. **5**(1): p. 84-90.
35. Doykos, P.M., et al., *Recommendations from a dialogue on evolving National Cancer Institute-designated comprehensive cancer center community outreach and engagement requirements: A path forward.* Health Equity, 2021. **5**(1): p. 76-83.
36. Kehrloesser, S., et al., *Analysing the attributes of comprehensive cancer centres and cancer centres across Europe to identify key hallmarks.* Mol Oncol, 2021. **15**(5): p. 1277-1288.
37. Mueller, N.M., et al., *The prevalence of dissemination and implementation research and training grants at National Cancer Institute-designated cancer centers.* JNCI Cancer Spectr, 2022. **6**(1).
38. Ringborg, U., et al., *The Porto European Cancer Research Summit 2021.* Mol Oncol, 2021. **15**(10): p. 2507-2543.
39. Kalra, M., et al., *Making national cancer institute-designated comprehensive cancer center knowledge accessible to community oncologists via an online tumor board: Longitudinal observational study.* JMIR Cancer, 2022. **8**(2): p. e33859.

40. Kirtane, K., et al., *Demographic disparities in receipt of care at a comprehensive cancer center.* Cancer Medicine, 2023. **12**(12): p. 13687-13700.
41. Lawler, M., et al., *European Groundshot-addressing Europe's cancer research challenges: A Lancet Oncology Commission.* Lancet Oncol, 2022.
42. Majumdar, D., C. Reynolds Kueny, and M. Anderson, *Impact of merging into a comprehensive cancer center on health care teams and subsequent team-member and patient experiences.* JCO Oncol Pract, 2022: p. OP2200280.
43. Alaniz, M. and T.R. Rebbeck, *The role of community outreach and engagement in evaluation of NCI Cancer Center Support Grants.* Cancer Causes & Control, 2023: p. 1-3.
44. Berns, A., *Academia and society should join forces to make anti-cancer treatments more affordable.* Molecular Oncology, 2024.
45. Fervers, B., et al., *An Integrated Cancer Prevention Strategy: the Viewpoint of the Leon Berard Comprehensive Cancer Center Lyon, France.* Cancer Prevention Research, 2024. **17**(4): p. 133-140.
46. Odedina, F.T., et al., *Operational strategies for achieving comprehensive cancer center community outreach and engagement objectives: impact and logic models.* Archives of Public Health, 2024. **82**(1): p. 35.
47. Ringborg, U., et al., *Strategies to decrease inequalities in cancer therapeutics, care and prevention: Proceedings on a conference organized by the Pontifical Academy of Sciences and the European Academy of Cancer Sciences, Vatican City, February 23–24, 2023.* Molecular Oncology, 2024. **18**(2): p. 245.
48. Schulmeyer, C.E., et al., *Improving the Quality of Care for Cancer Patients through Oncological Second Opinions in a Comprehensive Cancer Center: Feasibility of Patient-Initiated Second Opinions through a Health-Insurance Service Point.* Diagnostics, 2023. **13**(21): p. 3300.
49. Trapl, E.S., S. Koopman Gonzalez, and K. Austin, *A framework for building comprehensive cancer center's capacity for bidirectional engagement.* Cancer Causes & Control, 2024: p. 1-9.
50. Unger, J.M., et al., *National estimates of the participation of patients with cancer in clinical research studies based on Commission on cancer accreditation data.* Journal of Clinical Oncology, 2024: p. JCO. 23.01030.
51. World Health Organization, *Towards a strategy for cancer control in the Eastern Mediterrean Region*. 2009, Regional Office for Eastern Mediteranean: Cairo.
52. Bell, R., et al., *Cancerpedia*. 2018, Princess Margaret Cancer Center.
53. Oberst S, et al., *Comprehensive cancer care across the EU: Advancing the vision*. 2021: Brussels.
54. Jatho, A., S. Kibudde, and J. Orem. *An approach to comprehensive cancer control in low- and middle-income countires: The Ugandan Model*. 2022 [cited 2024 2nd July]; Available from: https://connection.asco.org/magazine/asco-international/approach-comprehensive-cancer-control-low-and-middle-income-countries.
55. McArthur, G., *VCCC Alliance submission to the Australian Government Department of the Treasury*. 2022, Victorian Comprehensive Cancer Centre Alliance: Melbourne.
56. Gospodarowicz, M., et al., *Cancer services and the comprehensive cancer center*, in *Disease control priorities*, H. Gelband, et al., Editors. 2015, The International Bank for Reconstruction and Development / The World Bank: Washington (DC). p. 195-210.
57. Alcantara, G. and N.J. Chao, *Administrative support*, in *The comprehensive cancer center: Development, integration and implementation*, M. Aljurf, et al., Editors. 2022, Springer: Switzerland. p. 85-91.
58. Aldehaim, M. and J. Phan, *Proposal for establishing a new radiotherapy facility*, in *The comprehensive cancer center: Development, integration and implementation*, M. Aljurf, et al., Editors. 2022, Springer: Switzerland. p. 41-55.

59. Babar, A. and A.J. Montero, *Building quality from the ground up in a cancer center*, in *The comprehensive cancer center: Development, integration and implementation*, M. Aljurf, et al., Editors. 2022, Springer: Switzerland. p. 3-13.
60. Chakraborty, R., N.S. Majhail, and J. Abraham, *Psychosocial and patient support services in comprehensive cancer centers*, in *The comprehensive cancer center: Development, integration and implementation*, M. Aljurf, et al., Editors. 2022, Springer: Switzerland. p. 93-106.
61. Chew, L. and M.C.M. Thum, *Pharmacy requirements for a comprehensive cancer center*, in *The comprehensive cancer center: Development, integration and implementation*, M. Aljurf, et al., Editors. 2022, Springer: Switzerland. p. 75-83.
62. Fleming, K.M., M. Klammer, and M.B.C. Koh, *Laboratory / pathology services and blood bank*, in *The comprehensive cancer center: Development, integration and implementation*, M. Aljurf, et al., Editors. 2022, Springer: Switzerland. p. 63-74.
63. Frith, J. and N.J. Chao, *Oncology nursing care*, in *The comprehensive cancer center: Development, integration and implementation*, M. Aljurf, et al., Editors. 2022, Springer: Switzerland. p. 57-62.
64. Grosso, D., M. Aljurf, and U. Gergis, *Building a comprehensive cancer center: Overall structure*, in *The comprehensive cancer center: Development, integration and implementation*, M. Aljurf, et al., Editors. 2022, Springer: Switzerland. p. 3-13.
65. Hashmin, S., et al., *Cancer management at sites with limited resources: challenges and potential solutions*, in *The comprehensive cancer center: Development, integration and implementation*, M. Aljurf, et al., Editors. 2022, Springer: Switzerland. p. 173-185.
66. Hussain, F., S. Alhayli, and M. Aljurf, *Data unit, translational reserach, and registries*, in *The comprehensive cancer center: Development, integration and implementation*, M. Aljurf, et al., Editors. 2022, Springer: Switzerland. p. 157-165.
67. Kharfan-Dabaja, M.A., *The infusion center*, in *The comprehensive cancer center: Development, integration and implementation*, M. Aljurf, et al., Editors. 2022, Springer: Switzerland. p. 35-40.
68. Kharfan-Dabaja, M.A., *The inpatient unit in a cancer center*, in *The comprehensive cancer center: Development, integration and implementation*, M. Aljurf, et al., Editors. 2022, Springer: Switzerland. p. 15-20.
69. Majhail, N.S. and M. De Lima, *Transplantation and cellular therapy*, in *The comprehensive cancer center: Development, integration and implementation*, M. Aljurf, et al., Editors. 2022, Springer: Switzerland. p. 121-33.
70. Murthy, H.S., R. Manochakian, and M.A. Kharfan-Dabaja, *Education and training*, in *The comprehensive cancer center: Development, integration and implementation*, M. Aljurf, et al., Editors. 2022, Springer: Switzerland. p. 167-72.
71. Shoemaker, L. and S. McInnes, *Starting a palliative care program at a cancer center*, in *The comprehensive cancer center: Development, integration and implementation*, M. Aljurf, et al., Editors. 2022, Springer: Switzerland. p. 107-120.
72. Wu, I.Q., F.L.W.I. Lim, and L.P. Koh, *Outpatient care*, in *The comprehensive cancer center: Development, integration and implementation*, M. Aljurf, et al., Editors. 2022, Springer: Switzerland. p. 21-33.
73. Yassine, F. and M.A. Kharfan-Dabaja, *Patient resources in a cancer centre*, in *The comprehensive cancer center: Development, integration and implementation*, M. Aljurf, et al., Editors. 2022, Springer: Switzerland. p. 145-155.
74. Rajan, A., *Assessing translational research excellence in European comprehensive cancer centres*. 2015, University of Twente: Netherlands.
75. Cancer Australia. *Australian Comprehensive Cancer Network*. 2024 [cited 2024 27th June]; Available from: https://www.canceraustralia.gov.au/key-initiatives/accn.

76. Czech Cancer Centre Network. *Criteria defining the status of Comprehensive Cancer Centre*. 2023 [cited 2023 18th September]; Available from: https://www.linkos.cz/english-summary/national-cancer-control-programme/czech-cancer-centre-network/criteria-defining-the-status-of-comprehensive-cancer-centre/.
77. CraNE. *European Network of Comprehensive Cancer Centres*. 2022 [cited 2023 November]; Available from: https://crane4health.eu/.
78. NCI, T.O.o.C.C.a. *NIH National Cancer Institute: Office of Cancer Centers*. 2023 [cited 2023 18th September]; Available from: https://cancercenters.cancer.gov/.
79. National Comprehensive Cancer Network. *National Comprehensive Cancer Network*. 2023 [cited 2023 18th Septembeer]; Available from: https://www.nccn.org/.
80. National Comprehensive Cancer Network. *Global Program*. 2024 [cited 2024 2nd July]; Available from: https://www.nccn.org/global/global-program.
81. Organisation European Cancer Institute. *OECI*. 2023 [cited 2023 18th September]; Available from: https://www.oeci.eu/.
82. German Cancer Aid, *Program for the development of interdisciplinary oncology centres of excellence in Germany: 10th call for applications*. 2023, German Cancer Aid: Germany.
83. Aljurf, M., et al., *The comprehensive cancer center: Development, integration and implementation*. 2022, Switzerland: Springer.
84. Lawler, M., et al., *European Groundshot-addressing Europe's cancer research challenges: A Lancet Oncology Commission.* Lancet Oncol, 2023.
85. National Cancer Institute. *NCI-Designated Cancer Centers*. 2023 [cited 2024 1st February]; Available from: https://www.cancer.gov/research/infrastructure/cancer-centers.
86. Aryanpour, Z., et al., *A single institution experience with papillary thyroid cancer: Are outcomes better at comprehensive cancer centers?* Am J Surg, 2021. **222**(4): p. 802-805.
87. Bilimoria, K.Y., et al., *Wait times for cancer surgery in the United States: trends and predictors of delays.* Ann Surg, 2011. **253**(4): p. 779-85.
88. Bristow, R.E., et al., *Impact of National Cancer Institute Comprehensive Cancer Centers on ovarian cancer treatment and survival.* J Am Coll Surg, 2015. **220**(5): p. 940-50.
89. Etzioni, D.A., et al., *Patient survival after surgical treatment of rectal cancer: impact of surgeon and hospital characteristics.* Cancer, 2014. **120**(16): p. 2472-81.
90. Freeman, A.T., et al., *Statewide geographic variation in outcomes for adults with acute myeloid leukemia in North Carolina.* Cancer, 2016. **122**(19): p. 3041-50.
91. Johnston, E.E., et al., *End-of-Life Intensity for Adolescents and Young Adults With Cancer: A Californian Population-Based Study That Shows Disparities.* J Oncol Pract, 2017. **13**(9): p. e770-e781.
92. Luchey, A.M., et al., *Change in Management Based on Pathologic Second Opinion Among Bladder Cancer Patients Presenting to a Comprehensive Cancer Center: Implications for Clinical Practice.* Urology, 2016. **93**: p. 130-4.
93. Merkow, R.P., et al., *National assessment of margin status as a quality indicator after pancreatic cancer surgery.* Ann Surg Oncol, 2014. **21**(4): p. 1067-74.
94. Rochigneux, P., et al., *Use of chemotherapy near the end of life: what factors matter?* Annals of Oncology, 2017. **28**(4): p. 809-817.
95. Shabihkhani, M., et al., *Metastatic Colorectal Cancer in Hispanics: Treatment Outcomes in a Treated Population.* Clin Colorectal Cancer, 2016. **15**(4): p. e221-e227.
96. Sultan, D.H., et al., *Minority use of a National Cancer Institute-Designated Comprehensive Cancer Center and non-specialty hospitals in two Florida regions.* Journal of Racial and Ethnic Health Disparities, 2015. **2**: p. 373-384.

97. Sun, C., et al., *Differential survival after prostate cancer by race: Role of NCI-designated comprehensive cancer centers.* Journal of Clinical Oncology, 2009. **27**(15_suppl): p. 6520-6520.
98. Wolfson, J., et al., *Impact of treatment site in adolescents and young adults with central nervous system tumors.* J Natl Cancer Inst, 2014. **106**(8): p. dju166.
99. Wolfson, J.A., et al., *Impact of care at comprehensive cancer centers on outcome: Results from a population-based study.* Cancer, 2015. **121**(21): p. 3885-93.
100. Colombet, I., et al., *Multicentre analysis of intensity of care at the end-of-life in patients with advanced cancer, combining health administrative data with hospital records: variations in practice call for routine quality evaluation.* BMC Palliat Care, 2019. **18**(1): p. 35.
101. Falcone, M., et al., *Evidence for racial/ethnic disparities in emergency department visits following breast cancer surgery among women in California: a population-based study.* Breast Cancer Res Treat, 2021. **187**(3): p. 831-841.
102. Fodero, R. and J. Bailey, *Comparing hospital costs and length of stay for cancer patients in New York State Comprehensive Cancer Centers versus nondesignated academic centers and community hospitals.* Health Serv Res, 2023. **58**(6): p. 1178-1188.
103. Freeman, A.T., et al., *Influence of Treating Facility, Provider Volume, and Patient-Sharing on Survival of Patients With Multiple Myeloma.* J Natl Compr Canc Netw, 2019. **17**(9): p. 1100-1108.
104. Nardi, E.A., et al., *Lung Cancer in Nonelderly Patients: Facility and Patient Characteristics Associated With Not Receiving Treatment.* Journal of the National Comprehensive Cancer Network J Natl Compr Canc Netw, 2019. **17**(8): p. 931-939.
105. Sandrucci, S., et al., *Different quality of treatment in retroperitoneal sarcomas (RPS) according to hospital-case volume and surgeon-case volume: a retrospective regional analysis in Italy.* Clin Sarcoma Res, 2018. **8**: p. 3.
106. Shaikh, H.G., et al., *Survival outcomes of post-transplant lymphoproliferative disorder: A 12-year analysis.* Journal of Clinical Oncology, 2021. **39**(15_suppl): p. e19543-e19543.
107. Shulman, L.N., et al., *Survival As a Quality Metric of Cancer Care: Use of the National Cancer Data Base to Assess Hospital Performance.* J Oncol Pract, 2018. **14**(1): p. e59-e72.
108. Wolfson, J., et al., *Adolescents and Young Adults with Acute Lymphoblastic Leukemia and Acute Myeloid Leukemia: Impact of Care at Specialized Cancer Centers on Survival Outcome.* Cancer Epidemiol Biomarkers Prev, 2017. **26**(3): p. 312-320.
109. Wolfson, J.A., et al., *Expenditures among young adults with acute lymphoblastic leukemia by site of care.* Cancer, 2021. **127**(11): p. 1901-1911.
110. European Commission, *Europe's Beating Cancer Plan: Communication from the commission to the European Parliament and the Council*. 2022, European Commission.
